# RNA degradome analysis reveals DNE1 endoribonuclease is required for the turnover of diverse mRNA substrates in Arabidopsis

**DOI:** 10.1093/plcell/koad085

**Published:** 2023-04-18

**Authors:** Vinay K Nagarajan, Catherine J Stuart, Anna T DiBattista, Monica Accerbi, Jeffrey L Caplan, Pamela J Green

**Affiliations:** Delaware Biotechnology Institute, University of Delaware, Newark, DE 19713-1316, USA; Delaware Biotechnology Institute, University of Delaware, Newark, DE 19713-1316, USA; Delaware Biotechnology Institute, University of Delaware, Newark, DE 19713-1316, USA; Delaware Biotechnology Institute, University of Delaware, Newark, DE 19713-1316, USA; Bio-Imaging Center, Delaware Biotechnology Institute, University of Delaware, Newark, DE 19713-1316, USA; Delaware Biotechnology Institute, University of Delaware, Newark, DE 19713-1316, USA

## Abstract

In plants, cytoplasmic mRNA decay is critical for posttranscriptionally controlling gene expression and for maintaining cellular RNA homeostasis. Arabidopsis DCP1-ASSOCIATED NYN ENDORIBONUCLEASE 1 (DNE1) is a cytoplasmic mRNA decay factor that interacts with proteins involved in mRNA decapping and nonsense-mediated mRNA decay (NMD). There is limited information on the functional role of DNE1 in RNA turnover, and the identities of its endogenous targets are unknown. In this study, we utilized RNA degradome approaches to globally investigate DNE1 substrates. Monophosphorylated 5′ ends, produced by DNE1, should accumulate in mutants lacking the cytoplasmic exoribonuclease XRN4, but be absent from DNE1 and XRN4 double mutants. In seedlings, we identified over 200 such transcripts, most of which reflect cleavage within coding regions. While most DNE1 targets were NMD-insensitive, some were upstream ORF (uORF)-containing and NMD-sensitive transcripts, indicating that this endoribonuclease is required for turnover of a diverse set of mRNAs. Transgenic plants expressing *DNE1* cDNA with an active-site mutation in the endoribonuclease domain abolished the *in planta* cleavage of transcripts, demonstrating that DNE1 endoribonuclease activity is required for cleavage. Our work provides key insights into the identity of DNE1 substrates and enhances our understanding of DNE1-mediated mRNA decay.

## Introduction

Controlling RNA stability is important for regulating gene expression. A major mechanism that governs RNA stability is cytoplasmic mRNA decay, which is responsible for the degradation of unwanted or aberrant transcripts. In plants and other eukaryotes, mRNA degradation is initiated by the progressive shortening of the poly(A) tail, predominantly by the CCR4–CAF1–NOT1 deadenylase complex (Collart 2003; Abbasi et al. 2013; Wahle and Winkler 2013; Webster et al. 2018). Deadenylated RNA can subsequently be degraded in the 3′ to 5′ direction by either the multisubunit RNA exosome complex or other exoribonucleases (e.g. SOV/DIS3L2; Zhang et al. 2010; Lubas et al. 2013; Malecki et al. 2013). Alternatively, a deadenylated transcript can be degraded in the 5′ to 3′ direction by the exoribonuclease XRN4, the cytoplasmic homolog of yeast and metazoan XRN1 (Kastenmayer and Green 2000; Souret et al. 2004; Nagarajan et al. 2019), after the 5′ cap is removed by the DCP1–DCP2–VCS decapping complex (Xu et al. 2006; Goeres et al. 2007; Iwasaki et al. 2007). In some cases, however, mRNA decapping can occur without the requirement of deadenylation (Badis et al. 2004; Conti and Izaurralde 2005). Additionally, some mRNAs undergo co-translational decay when ribosomes are stalled or paused, a process that requires XRN1/XRN4 (Pelechano et al. 2015; Yu et al. 2016; Carpentier et al. 2020; Tuck et al. 2020). RNA degradation is also initiated by endoribonucleases (endoRNases) that internally cleave RNA molecules via hydrolysis to produce unprotected 5′ and 3′ fragments that are rapidly eliminated by cellular exoribonucleases (Tomecki and Dziembowski 2010; Schoenberg 2011). In plants and other eukaryotes, endoRNases cleave specific RNA substrates to repress gene expression and are therefore integral to various RNA decay pathways. Examples of these include miRNA-directed RNA cleavage by AGO proteins (Fagard et al. 2000; Llave et al. 2002; Baumberger and Baulcombe 2005; Jones-Rhoades et al. 2006), tRNA splicing endonuclease-initiated mRNA decay (TED; Hurtig et al. 2021), regulated IRE1-dependent decay (RIDD; Hollien et al. 2009; Mishiba et al. 2013), and nonsense-mediated mRNA decay (NMD) in metazoans (Gatfield and Izaurralde 2004; Huntzinger et al. 2008; Eberle et al. 2009).

In eukaryotic cells, NMD targets a broad range of mRNAs to control gene expression. In addition to defective mRNAs with premature termination codons (PTCs) arising from splicing or transcription errors, NMD also targets faithfully produced mRNAs to regulate their overall expression. Transcripts with particular features, such as upstream open reading frames (uORFs), long 3′ UTRs (usually ≥350 nt), and introns downstream of stop codons, are often enriched among NMD targets (Kertész et al. 2006; Kerényi et al. 2008; Popp and Maquat 2013; He and Jacobson 2015). During translation, the highly conserved RNA helicase and NMD factor UPF1 is recruited to targeted mRNA–protein complexes where it undergoes phosphorylation and initiates degradation of the NMD-sensitive mRNA. In yeast and plants, NMD-sensitive transcripts are turned over via decapping followed by 5′ to 3′ degradation by XRN1/XRN4 and/or deadenylation followed by 3′ to 5′ degradation by the RNA exosome (Nagarajan et al. 2013; Shaul 2015). In metazoans, the endoRNase SMG6 cleaves NMD-sensitive transcripts near stop codons, and the resulting mRNA fragments are further degraded by decapping and/or deadenylation followed by exoribonucleolytic activity (Chen and Shyu 2003; Lejeune et al. 2003; Lloyd 2018; Karousis and Mühlemann 2019). Transient assays in *Nicotiana benthamiana* expressing a PTC-containing reporter showed lack of detectable 3′ cleavage products, indicating that plants may not utilize an endoRNase to cleave NMD targets (Mérai et al. 2012).

Arabidopsis UPF1 interacts with an endoRNase in an RNA-dependent manner (Chicois et al. 2018). Recently, the same endoRNase was also found to physically interact with the decapping factor DCP1 and was hence named DCP1-ASSOCIATED NYN ENDORIBONUCLEASE 1 (DNE1, AT2G15560; Schiaffini et al. 2022). In our previous Arabidopsis RNA degradome study, which globally captured partially degraded 5′ monophosphorylated (5′P) RNA, we observed that some endogenous NMD-sensitive transcripts are cleaved by an unknown endoRNase, and the resultant 3′ RNA fragments are degraded by XRN4 (Nagarajan et al. 2019). Whether or not DNE1 targets NMD-sensitive or insensitive mRNAs remains to be determined. Arabidopsis DNE1 contains an Nedd4-BP1 YacP Nuclease (NYN) endoRNase domain and is a homolog of metazoan Meiosis Arrest Female 1 (MARF1). The NYN domain typically consists of 4 conserved aspartic acid (Asp, D) residues that chelate a single divalent cation (Mg^2+^ or Mn^2+^) in a similar way as the PilT N-terminal (PIN) domain (Anantharaman and Aravind 2006) present in SMG6. The N-terminal NYN domain of MARF1 cleaves single-stranded RNA (Nishimura et al. 2018; Yao et al. 2018), while the C-terminal Limkain, Oskar, and Tudor domain (LOTUS/OST-HTH) binds RNA targets (Nishimura et al. 2018; Yao et al. 2018; Brothers et al. 2020). Human MARF1 also physically interacts with the DCP1–DCP2 decapping complex (Nishimura et al. 2018). Interestingly, Arabidopsis DNE1 interacts only with DCP1 and not with DCP2 (Schiaffini et al. 2022), indicating that different RNA decapping subcomplexes may exist in the cell. Therefore, the evolutionarily conserved relationship between DNE1 and the mRNA decapping complex could be crucial for mRNA decay. In Arabidopsis, the loss of DNE1 in a decapping mutant background led to defective phyllotaxy (Schiaffini et al. 2022). Another *DNE1* mutant was shown also to have altered root growth during osmotic stress (Luhua et al. 2013), while a DNE1 overexpression line displayed more tolerance to oxidative stress (Luhua et al. 2008), thereby implicating DNE1 in different aspects of plant development and stress responses.

To elucidate the function of DNE1 in plant mRNA decay, it is crucial to identify its substrates and determine how it contributes to RNA turnover on a global scale. Here, we identified endogenous targets of Arabidopsis DNE1 using RNA degradome approaches that capture RNA substrates. Using a strategy similar to that of our previous studies that identified targets of human SMG6 (Schmidt et al. 2015) and yeast Sen2 (Hurtig et al. 2021), we utilized the RNA degradome approaches Parallel Analysis of RNA Ends (PARE, German et al. 2008) and Genome-wide Mapping of Uncapped and Cleaved Transcripts (GMUCT, Gregory et al. 2008; Willmann et al. 2014) to capture 5′P RNAs that are sensitive to degradation by the Arabidopsis 5′ to 3′ exoribonuclease XRN4. Comparing these potential endoRNase cleavage events in plants with and without DNE1 function identified 5′P cleavage sites that are dependent on this endoRNase. Our work allowed us to gain insights on the mRNA substrates targeted by this endoRNase, thereby revealing the molecular mechanism of DNE1-dependent mRNA decay. Importantly, this study is the first to globally identify endogenous cleaved targets of a MARF1 family member in eukaryotes.

## Results

### Features of Arabidopsis DNE1 support its role as a cytoplasmic endoRNase

**Figure 1.**
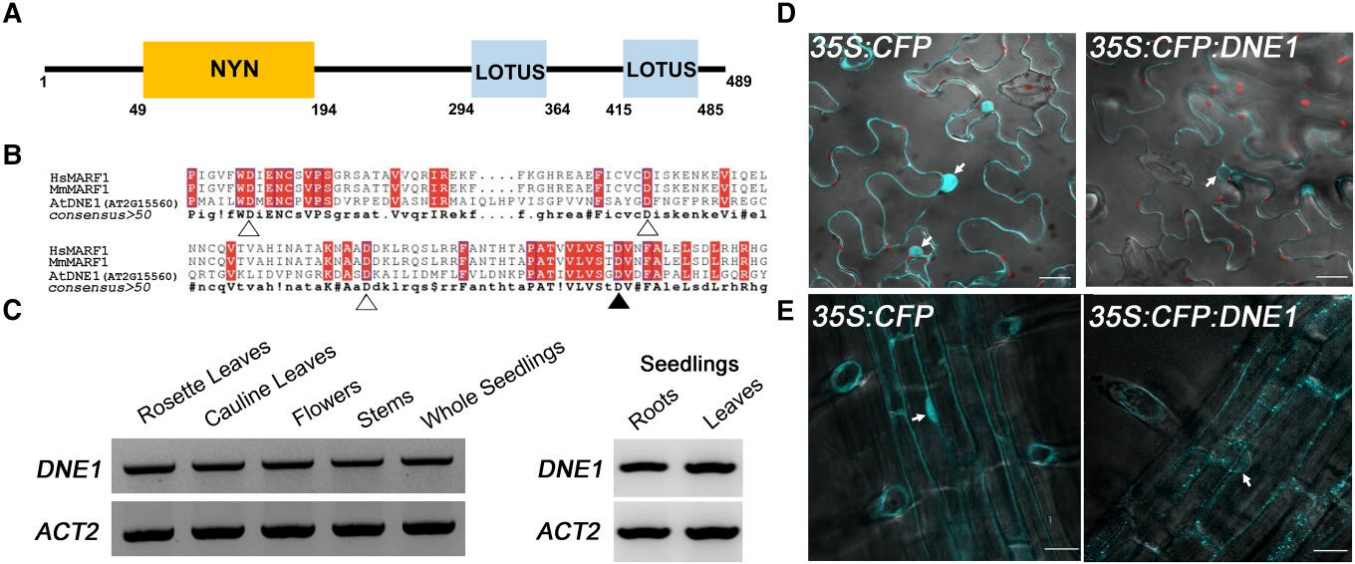
Arabidopsis DNE1 is an NYN domain-containing protein that localizes to the cytoplasm. **A)** Schematic illustration of the domain architecture of Arabidopsis DNE1. NYN, endoribonuclease domain; LOTUS, RNA-binding domain. **B)** Amino acid sequence alignment of the NYN domains of Arabidopsis DNE1 and metazoan MARF1. Shaded boxes, conserved residues. Triangles, residues required for ssRNase activity (Yao et al. 2018); filled triangle, D153 residue used in subsequent mutational analysis. Hs, human; Mm, mouse; At, Arabidopsis. **C)** RT-PCR results showing the *DNE1* and *ACT2* genes are expressed in each of the different Arabidopsis organs tested. The location of primers (6958 and 6959) amplifying 1 kb region of the 1.47 kb *DNE1* CDS is shown in Supplemental Fig. S1. RNA was isolated from two-week-old seedlings grown on solid germination media and six- to eight-week-old adult plants grown in soil. Gel images are representative of three biological replicates. **D)** Transient expression of CFP and CFP:DNE1 in *N. benthamiana* leaves captured on an LSM880 multiphoton confocal microscope. Scale bars, 25 *μ*m. **E)** Stable expression of CFP and CFP:DNE1 in transgenic Arabidopsis roots captured on a Dragonfly spinning disk confocal microscope. Images are representative of two independent T2 lines. Scale bars, 40 *μ*m. For **D)** and **E)**, overlaid images of fluorescence and differential interference contrast (DIC) are shown as a single slice from a z-stack maximum intensity projection. Arrows, selected nuclei in individual epidermal cells.

It has previously been shown that DNE1 interacts with the critical NMD effector UPF1 as well as major components of the 5′ to 3′ decay machinery (Chicois et al. 2018; Schiaffini et al. 2022). Therefore, we sought to understand the function of DNE1 in plant mRNA decay. DNE1 contains an N-terminal NYN domain and two C-terminal LOTUS domains (Fig. 1A). Importantly, DNE1 is most closely related to the well-characterized metazoan MARF1, though it contains fewer LOTUS domains (Schiaffini et al. 2022). The LOTUS domain of human MARF1 has been recently shown to bind with high affinity to G-rich RNAs, particularly RNAs with G4 tertiary structure (Ding et al. 2020). Ribonuclease assays have demonstrated that MARF1 cleaves its RNA targets via its catalytic NYN domain (Nishimura et al. 2018; Yao et al. 2018). Specifically, it has been shown that four distinct aspartic acid (Asp, D) residues within the NYN domain of mouse MARF1 (D178, D215, D246, and D272) are required for ssRNA cleavage (Yao et al. 2018). To determine whether DNE1 contains the essential Asp residues required for endoRNase activity, we generated a multiple sequence alignment of the NYN domains of DNE1, human MARF1, and mouse MARF1 (Fig. 1B). The arrangement of the amino acid residues within the NYN domains of these proteins was remarkably similar. Moreover, the Asp residues required for ssRNA cleavage are conserved in DNE1, so we conclude that Arabidopsis DNE1 is likely an active endoRNase (Fig. 1B).

To characterize *DNE1* expression in Arabidopsis, we examined *DNE1* mRNA abundance in several above-ground organs. *DNE1* expression was evaluated in the rosette leaves, cauline leaves, flowers, and stems of adult plants, as well as in the roots and leaves of seedlings. The results indicate that *DNE1* is expressed in all Arabidopsis organs examined (Fig. 1C). To verify the location of DNE1 in individual cells, the full-length coding region of *DNE1* was fused in-frame at the C-terminal end of cyan fluorescent protein (CFP) under the control of the constitutive cauliflower mosaic virus 35S promoter. The *35S:CFP:DNE1* localization construct and the *35S:CFP* empty vector were transformed into *Agrobacterium tumefaciens*; then, both constructs were transiently transformed into *N. benthamiana* leaves and stably transformed into Arabidopsis. Our analysis following transient (Fig. 1D) and stable (Fig. 1E) transformation shows that CFP:DNE1 localizes to distinct foci mostly in the cytoplasm, independently confirming and expanding upon previously published transient assays (Chicois et al. 2018). We observed CFP:DNE1 both in the cytoplasm and cytosolic foci in the root cells of transgenic plants (Fig. 1E). Other than a very faint signal in the nucleus, which can be explained by cytosol around the nuclear periphery, most of the CFP:DNE1 expression was observed in the cytoplasm. These findings support the results from an earlier study, which showed that DNE1 localizes to processing bodies (P-bodies) or cytoplasmic foci enriched in mRNA degradation factors (Schiaffini et al. 2022). Overall, these analyses suggest that DNE1 may function as an endoRNase in mRNA decay.

### Identification of DNE1-dependent 5′P sites within mRNAs across the transcriptome

To obtain insight into the role of DNE1 in mRNA decay, we sought to characterize the distribution of DNE1-dependent cleavage sites throughout the transcriptome. RNA degradome approaches such as PARE and GMUCT have been used to globally identify populations of uncapped RNA by capturing 5′P RNA, which can be produced by endoRNase cleavage or decapping. The 3′ RNA fragments produced by the action of PIN and related NYN domain-containing endoRNases carry a 5′P terminus (Cook et al. 2013; Boehm et al. 2014; Matelska et al. 2017; Senissar et al. 2017), and the RNA degradome approach is ideally suited to capture such RNA molecules and subsequently map the locations of these sites. We have used RNA degradome approaches previously to successfully map 5′P cleavage sites of the PIN domain-containing endoRNase SMG6 after reducing its transcript abundance via RNAi (Schmidt et al. 2015). Therefore, to characterize Arabidopsis DNE1 and study the substrates of this enzyme, we identified a SALK T-DNA line disrupting the *DNE1* gene with the insertion within its coding sequence (CDS, at position 901 nt; Supplemental Fig. S1A), henceforth referred to as *dne1* [same as the *dne1-1* mutant allele in Schiaffini et al. (2022)]. In this line, *DNE1* expression was not detectable (Supplemental Fig. S1B), and primers downstream of the T-DNA insertion site confirmed that *DNE1* transcript levels were strongly compromised (discussed later, Supplemental Fig. S9A).

We next sought to stabilize and identify cleaved 3′ RNA fragments that could be generated by DNE1 activity. To enhance the detection of such decay intermediates, *dne1* was crossed with *xrn4-5*, a null mutant of the major cytoplasmic 5′ to 3′ exoribonuclease XRN4 (Souret et al. 2004). We identified *dne1 xrn4* double mutants by testing the expression of both *DNE1* and *XRN4* in the F2 progeny (Supplemental Fig. S1B). To capture DNE1-dependent 5′P sites, we generated GMUCT and PARE libraries from poly(A)^+^ RNA isolated from seedlings and rosette leaves, respectively, and compared the RNA decay profiles of Col-0, *xrn4*, and *dne1 xrn4*. We generated the initial degradome libraries using the PARE method, which captures 20 nt sequences; subsequently, we adopted GMUCT to increase our confidence in detecting RNA decay events, as it allows for the sequencing of longer (50 nt) degradome reads (Supplemental Table S1). We used poly(A)^+^ RNA to generate the PARE and GMUCT libraries, since our previous analysis that separately compared poly(A)^+^ and poly(A)^−^ RNA fractions showed that the majority of endoribonucleolytic cleavages were identified in the poly(A)^+^ RNA fraction (Nagarajan et al. 2019). This is consistent with the observation that such cleavage events and their downstream intermediates appear to bypass deadenylation (Stevens et al. 2002; Gatfield and Izaurralde 2004; Doma and Parker 2006; Eberle et al. 2009; Boehm et al. 2014; Lykke-Andersen et al. 2014).

**Figure 2.**
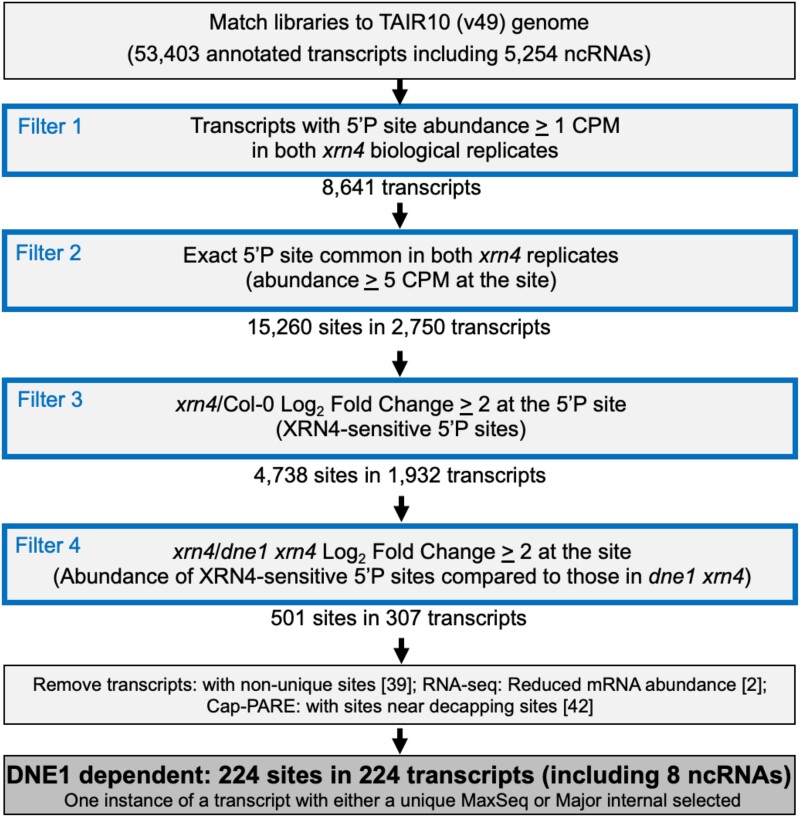
Computational pipeline for identifying DNE1 cleavage sites from seedling GMUCT libraries. Output is shown in bold font within a darker shaded box. Cleavage site corresponds to the position of the 5′P terminus of either a DNE1-dependent MaxSeq or a Major internal on a transcript. MaxSeq is the most abundant sequence on a transcript. Major internal is the next most abundant sequence on a transcript when the most abundant sequence is at the decapped site. The data are filtered (Filters 1 to 4) using the criteria described in the Materials and methods. Numbers in [ ] indicate transcripts that did not pass the filter. Additional details of the filters used in this analysis and the fold-change distribution are in Supplemental Fig. S3.

Using a computational pipeline similar to the one used for identifying targets of the Sen2 endoRNase (Hurtig et al. 2021), we identified DNE1 cleavage sites that are XRN4-sensitive (Fig. 2 and Supplemental Data Set S1). We hypothesized that DNE1-dependent 5′P sites with increased abundance in *xrn4* should be absent or drastically reduced in *dne1 xrn4*, when the endoRNase (DNE1) cleaving at that site is absent. Based on previous studies, we would expect *xrn4* libraries to contain more 5′P sites than Col-0, since XRN4 is required for the turnover of most cytosolic decay intermediates with a 5′P terminus. By plotting the abundance (CPM ≥ 1) pairwise between replicates (Pearson's *r* ≥ 0.94; *R*^2^ ≥ 0.89) of Col-0, *xrn4*, and *dne1 xrn4* seedling GMUCT libraries for each set of independent biological experiments, we found our data to be reproducible (Supplemental Fig. S2). We saw a similar pattern of reproducibility between the biological replicates from the PARE method (Pearson's *r* ≥ 0.90; *R*^2^ ≥ 0.80) of Col-0, *xrn4*, and *dne1 xrn4* rosette leaf samples (Supplemental Fig. S2). Next, for all 5′P sites with an abundance of CPM ≥ 1 or CPM ≥ 5, we compared the fold changes (FCs) for *xrn4*/Col-0, *xrn4/dne1 xrn4*, and *dne1 xrn4*/Col-0 (Supplemental Fig. S3A). We found consistent patterns of fold-change distribution between the replicates for all 3 comparisons. Interestingly, the FCs between *xrn4*/Col-0 were mostly comparable with *dne1 xrn4*/Col-0, suggesting that the loss of DNE1 does not dramatically impact the RNA degradome. In line with this notion, while the fold-change comparison of *xrn4/dne1 xrn4* was consistent, the overall FCs were smaller than those seen with *xrn4*/Col-0 and *dne1 xrn4/*Col-0. Based on these results, we infer that DNE1 targets are fewer and could be a specific set of mRNAs. Next, we identified XRN4-sensitive 5′P sites (*N* = 4,738 from 2,750 transcripts) that overaccumulate (CPM ≥ 5, log_2_ FC ≥ 2) in *xrn4* compared with Col-0 (Fig. 2). We then used the filtered XRN4-sensitive 5′P sites to select for those that show a log_2_ FC ≥ 2 increase in abundance in *xrn4* compared with *dne1 xrn4*. Using these stringent cutoffs, our pipeline identified 501 prominent XRN4-sensitive 5′P sites that rely on DNE1 activity (Fig. 2).

In order to eliminate decapping sites that could be incorrectly identified as cleavage sites, we took advantage of our previous data generated using Cap-PARE (C-PARE; Supplemental Data Set S2), which experimentally verified decapping sites in Arabidopsis (Nagarajan et al. 2019). Therefore, for the cleavage site analysis, we removed 5′P sites that were near the annotated transcription start site (TSS) or experimentally verified decapping site (Nagarajan et al. 2019). Since we had RNA-seq data for seedlings, we evaluated transcript abundances to ensure that reduced abundance at a 5′P site in *dne1 xrn4* was not due to lower expression of the full-length target mRNA compared with *xrn4* (Supplemental Data Sets S1 and S4). Additionally, sequences that were not unique and appeared in the genome more than once were not included. While in some cases we found multiple DNE1-dependent 5′P sites within a transcript, for the sake of simplicity, we restricted our analysis to the most abundant DNE1-dependent 5′P site per transcript. In this process, we identified two key distinct types of 5′P sites that were mutually exclusive and occurred at 1 site per target transcript: (i) the MaxSeq, the most abundant 5′P site (and sequence) within the transcript, and (ii) the Major internal, the second most abundant 5′P site (and sequence) when the decapping site was the most abundant. In seedlings, we identified 154 MaxSeq and 70 Major internal sites from as many transcripts (Fig. 2); these 224 5′P sites showing DNE1-dependent accumulation in *xrn4* seedlings were classified as DNE1 cleavage sites (Supplemental Data Set S1). The transcripts that are cleaved by DNE1 at these sites were classified as DNE1 targets. We compared mRNA abundances (RNA-seq) of DNE1 targets to see if these transcripts accumulate differentially between *dne1 xrn4* and *xrn4*. Surprisingly, there was very little difference in full-length transcript abundances of DNE1 targets between the *xrn4* and *dne1 xrn4* (Supplemental Fig. S3B). We inferred the following from this result: first, the 3′ RNA fragment abundance in *xrn4* does not contribute to an overall change in transcript abundance as determined by RNA-seq. Second, there is no compensatory increase in full-length abundances of DNE1 targets when DNE1 is absent, consistent with the lack of impact of DNE1 loss on global gene expression observed in this study (Supplemental Data Set S4) and in an earlier study (Schiaffini et al. 2022).

Using the same computational pipeline for the leaf PARE libraries, we identified 74 MaxSeq and Major internal sites (37 each) from as many transcripts that showed DNE1-dependent accumulation (Supplemental Fig. S4 and Data Set S3). These 5′P sites could not be filtered for full-length transcript abundance differences between *xrn4* and *dne1 xrn4* since we lacked RNA-seq data for leaf. Of the 74 DNE1 targets identified in the leaf samples, 34 (Supplemental Data Set S3) overlapped with the seedlings (*P* < 0.001; Fisher’s exact test). There were 24 DNE1 cleavage sites that were at the exact position in both, indicating that DNE1 cleaves these target mRNAs at the same site in both young seedlings and mature leaves.

### Patterns of DNE1 cleavage sites within transcripts

**Figure 3.**
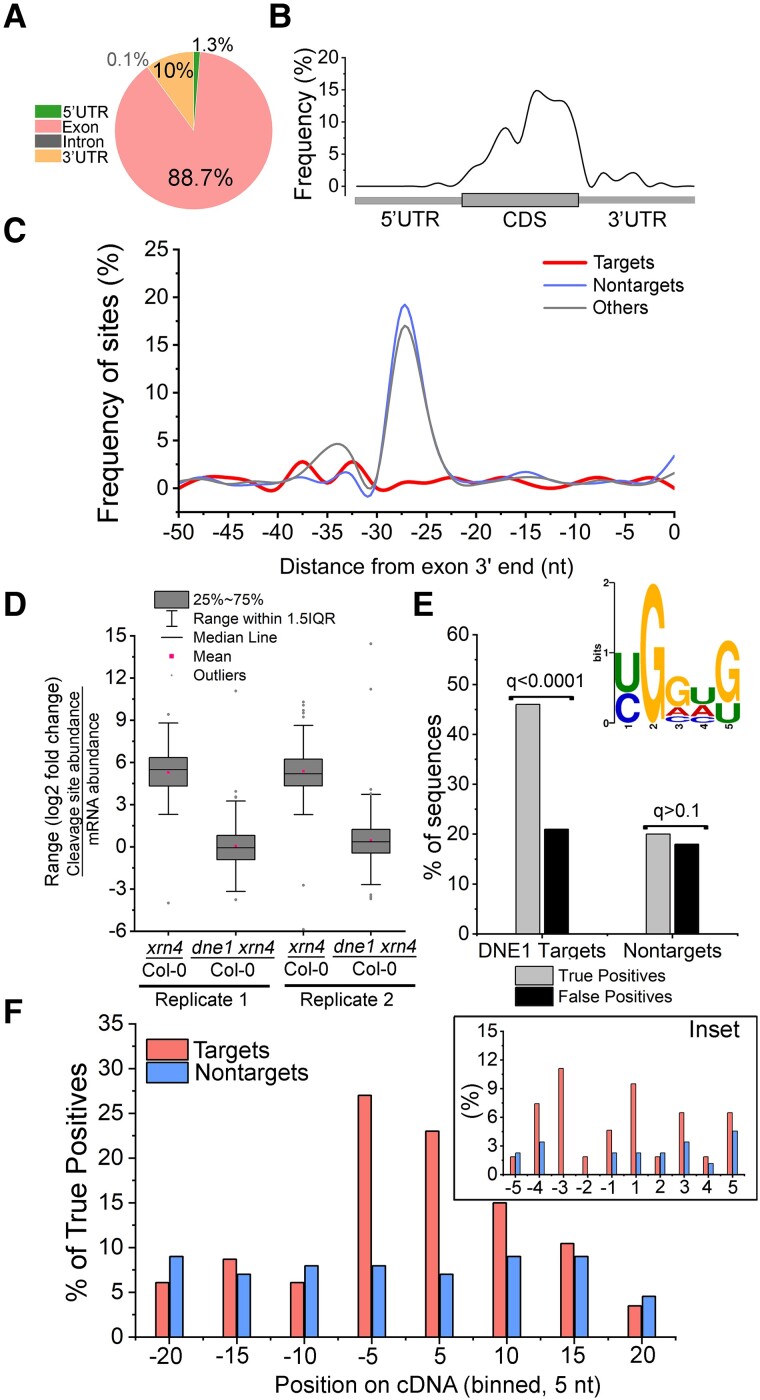
Features of DNE1 cleavage sites identified from seedling GMUCT analysis. **A)** DNE1 cleavage sites predominantly occur within exons. Pie chart showing the percentage of DNE1 cleavage sites (MaxSeq and Major internal) within distinct RNA regions. Cleavage sites are described in Fig. 2. **B)** Metagene analysis showing distribution of DNE1 cleavage sites across the length of mRNA transcripts. The regions (5′ UTR, CDS, and 3′ UTR) from TAIR10 mRNAs were individually binned spanning 1% of their total length, and the occurrence of cleavage sites within each bin was determined. **C)** DNE1 cleavage sites are not enriched near exon–exon junctions. Distribution of the relative frequency of cleavage site (targets, *N* = 185 sites in CDS) occurrences in the 50 nt region upstream of an exon–exon junction. The frequency of 5′P site occurrences within nontarget and other transcripts is also shown. **D)** DNE1 regulates cleavage site abundance of target transcripts. FC of cleavage site abundance (GMUCT) compared with FC of mRNA abundance (RNA-seq) for DNE1 target transcripts (Supplemental Data Set S1). **E)** Enrichment of a G-rich YGGWG sequence near DNE1 cleavage site. The YGGWG motif logo was generated using the MEME motif discovery tool (MEME Suite 5.5.0) using the Differential Enrichment function (*E* = 0.001) by comparing DNE1 target sequences against a control set (see description below). Histogram bars, enrichment of YGGWG motif shown as a percentage of true and false positives for DNE1 targets and nontarget sequences using the SEA analysis (MEME suite). *Q*-values are FDR-adjusted *P*-values. **F)** Distribution of G-rich sequences near DNE1-dependent 5′P sites. Position of YGGWG occurrences among true positive sequences (DNE1 target and nontarget) from **E**. A window (bin size = 5 nt) flanking the 5′P site (at position 1) is shown. Inset, frequency of YGGWG occurring within a 5-nt (bin size = 1 nt) sequence on either side of 5′P site (at position 1). For **C)** and **E)**, data sets were targets, transcripts that contain DNE1 cleavage sites as described in Fig. 2; nontargets, transcripts that contain 5′P sites with no change in accumulation between *xrn4* and *dne1 xrn4* (log_2_ FC ≥ −0.25 and ≤0.25; *N* = 486); and others, XRN4-insensitive transcripts that contain 5′P sites with no change in accumulation between *xrn4* and Col-0 (log_2_ FC ≥ −0.25 and ≤ 0.25; *N* = 3,214) and excludes targets and nontargets. For the MEME analysis, XRN4-sensitive sequences (*xrn4*/Col-0 log_2_ FC > 1; *N* = 1,186) were used as a control set. Only 5′P sites within CDS of nuclear-encoded protein-coding transcripts that did not overlap with decapped sites were included in the analysis.

We examined the distribution of DNE1 cleavage sites within different regions of the transcriptome. The sites mostly occurred within intragenic regions, and of these, the majority occurred in exons (89%) and 3′ UTRs (10%), and considerably fewer occurred in 5′ UTRs and introns (Fig. 3A). This trend was similar in leaves, with 84% and 16% in exons and 3′ UTRs, respectively. Next, we sought to determine whether the DNE1 cleavage sites are preferentially found in particular regions of mRNA transcripts. For this purpose, metagene analysis was performed. The DNE1 cleavage sites were predominantly found near the 3′ end of the CDS and start of the 3′ UTR in both seedling (Fig. 3B) and leaf (Supplemental Fig. S5A) RNA degradomes, albeit slightly more proximal to the stop codon in the latter. These results indicate that DNE1 cleavage sites are mostly within the CDS and cluster close to the stop codon in target mRNAs.

We next examined where within the exons DNE1 cleavage sites occurred. To evaluate this in more detail, we included 2 different controls in the analysis: (i) nontargets, transcripts that contain 5′P sites with no change in accumulation between *xrn4* and *dne1 xrn4*, and (ii) others, XRN4-insensitive transcripts that contain 5′P sites with no change in accumulation between *xrn4* and Col-0. In plants, *Caenorhabditis elegans*, and humans, 5′P sites are enriched upstream of exon–exon junctions (Lee et al. 2020). During splicing of transcripts, a multiprotein Exon Junction Complex (EJC) is deposited 20 to 24 nt upstream of each exon–exon boundary (Hir et al. 2016). It has been proposed that the increased occurrence of 5′P sites, especially between 25 and 30 nt upstream of an exon–exon junction, is due to EJC protection of 5′ ends from RNA degradation (Lee et al. 2020). As expected, there was an increased occurrence of 5′P sites among nontarget and XRN4-insensitive transcripts between 25 and 30 nt upstream of the junction, with a discernable peak ∼27 nt (Fig. 3C). In contrast, the frequency of DNE1 cleavage sites was drastically reduced in this region (Fig. 3C). A similar pattern was also observed in the leaf samples, wherein very few DNE1 cleavage sites were found upstream of an exon–exon junction compared with nontargets (Supplemental Fig. S5B). Therefore, our results indicate that DNE1 cleavage sites in both leaf and seedlings are not associated with exon–exon junctions.

We investigated the effects of DNE1 on cleavage site abundance in the seedling RNA degradome. We compared the fold-change distributions between cleavage site abundance (GMUCT) and transcript abundance (RNA-seq) for DNE1 targets between *xrn4/*Col-0 and *dne1 xrn4*/Col-0 (*N* = 199). Clearly, the cleavage site abundances were dramatically reduced in *dne1 xrn4* compared with *xrn4*, indicating that DNE1 elevates the cleavage site abundance of target transcripts (Fig. 3D). We then analyzed the cleavage site target sequences to determine if there is any sequence preference displayed by DNE1. For this purpose, we assayed a 40-nt sequence spanning the DNE1 cleavage site to test for short enriched sequences using the MEME suite. Control sequences containing XRN4-sensitive sites within the CDS were used to determine DNE1-specific effects at the cleavage sites. DNE1 target sequences were enriched for a degenerate G-rich sequence, YGGWG [where Y, (C/U); W, (A/U); *E* = 0.001; Fig. 3E and Supplemental Data Set S5]. While 50% of DNE1 target sequences were significantly positive for this G-rich motif, the occurrence of this motif in nontarget sequences was comparable with that found in the control sequences (Fig. 3E). Further, we mapped the location of this motif along the 40 nt region spanning the 5′P site for the positive sequences identified in Fig. 3E. In almost 51% of DNE1 target sequences, YGGWG clustered within 5-nt flanking the 5′P site, whereas this motif occurred in 14% of nontarget sequences in this region (Fig. 3F). These results indicate that this G-rich motif occurs in close proximity to the DNE1 cleavage site. Our analysis suggests that YGGWG could be specific to RNA sequences that are targeted and/or recognized by DNE1.

We also analyzed the DNE1 targets for overrepresented gene ontology (GO) terms to identify novel associations of this endoRNase. There were several GO terms of general cellular processes that were significantly overrepresented among DNE1 targets (Supplemental Table S3). The top category was “Glucosinolate metabolism,” and products of this plant secondary metabolic pathway play roles in plant defense response toward insects. Another major overrepresented category “Regulation of RNA metabolic process” includes several transcription regulators, specifically those implicated in a variety of plant stress responses (e.g. *MYB73/AT4G37260*, *HB6/AT2G22430*, *HBI1/AT2G18300*, *BEE2/AT4G36540*, and *MYBR1/AT5G67300*). Gene products of “Response to ABA” were also overrepresented and included *ABI1/AT4G26080*, a major negative regulator of ABA signaling, suggesting a role for DNE1 in plant ABA responses. Although not specifically overrepresented, there were several developmental genes known to be important for leaf and flower morphogenesis (e.g. *RPL/AT5G02030*, *NGA1/AT2G46870*, *MAF3/AT5G65060*, and *OVA5/AT3G13490*); therefore, we speculate that DNE1 is required for controlling the levels of some of these gene products. It is noteworthy that DNE1 targets include 2 well-studied circadian regulators, TIME FOR COFFEE (*TIC*/*AT3G22380*) and PSEUDO RESPONSE REGULATOR 7 (*PRR7/AT5G02810*). In plants, TIC is important for maintaining the amplitude and timing of clock gene expression and integrates developmental, metabolic, and stress signals (Hall et al. 2003; Ding et al. 2007). Perhaps, DNE1 has a role in the posttranscriptional control of *TIC* expression and plant clock function.

### Relationship between DNE1 targets, NMD, and other RNA decay pathways

**Figure 4.**
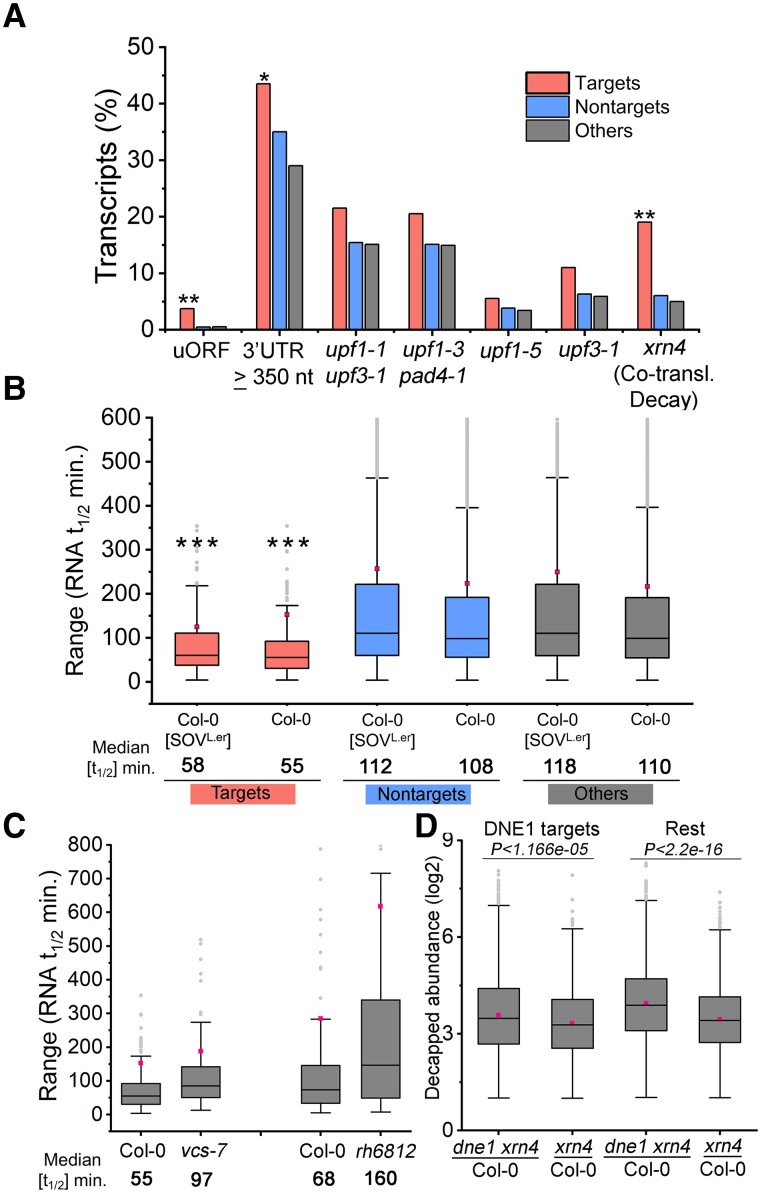
Characteristics of DNE1 target transcripts. **A)** The association of DNE1 target transcripts with NMD. Transcript features: uORF-containing transcripts; 3′ UTR lengths (TAIR10); transcripts elevated in NMD mutant seedlings (*upf1-1 upf3-1*, Drechsel et al. 2013; *upf1-3 pad4-1*, Raxwal et al. 2020; and *upf1-5* and *upf3-1*, Degtiar et al. 2015); co-translational decay targets of XRN4 (Carpentier et al. 2020). *P*-values (**P < 0.001; *P < 0.01) were determined by the Fisher’s exact test. **B)** Most DNE1 targets are unstable transcripts. Box plots showing the range of estimated RNA *t*_1/2_ for DNE1 targets. Decay rates and median RNA *t*_1/2_ values were obtained from Sorenson et al. (2018). *P*-values (***P < 2.2E−16) were determined by the Wilcoxon test. **C)** Impact of VCS and DDH1/DDX6-like loss on DNE1 target mRNA half-lives. Box plots showing the range of estimated RNA *t*_1/2_ for DNE1 targets in *vcs-7* and *rh6812* (DDH1/DDX6-like triple mutant). Decay rates and median RNA *t*_1/2_ values were obtained from *vcs-7* (Sorenson et al. 2018) and *rh6812* (Chantarachot et al. 2020). **D)** Influence of DNE1 on abundance of XRN4-sensitive decapped substrates. Range of relative abundances at decapping sites in *dne1 xrn4* and *xrn4* compared with those in Col-0. Decapped positions were extracted from seedling Cap-PARE libraries of Col-0 and *xrn4* (Nagarajan et al. 2019). Transcripts in *dne1 xrn4* and *xrn4* with minimum abundance ≥5 CPM at the decapping site with *xrn4*/Col-0 log_2_ FC ≥1 were considered. DNE1 targets (*N* = 73) and all other transcripts (rest, *N* = 1,148) that overaccumulate an XRN4-sensitive decapped intermediate were analyzed. *P*-values were determined by the Wilcoxon test. For **A)** and **B)**, data sets were as described in the legend of Fig. 3. For **B** to **D)**, box plots are as described in Fig. 3.

Given the previously reported interaction between DNE1 and the NMD effector UPF1, we tested whether NMD-sensitive transcripts were also DNE1 targets. For this purpose, we used multiple public genome-wide gene expression data sets of NMD mutant seedlings. These include 2 major studies that utilized different *upf1* mutants for identifying NMD targets. In the first study, *upf1-1 upf3-1*, which is deficient in 2 critical NMD factors, was shown to impact many NMD-sensitive transcripts (Drechsel et al. 2013). In the second study, *upf1-3 pad4-1*, which suppresses some of the severe pathogen responses of the stronger mutant allele of *upf1*, allowed for the detection of NMD-specific effects on the transcriptome (Raxwal et al. 2020). Of the 224 DNE1 targets, we found that 46 and 44 transcripts were elevated in *upf1-1 upf1-3* and *upf1-3 pad4-1*, respectively, indicating that ∼21% of DNE1 targets could be NMD-sensitive (Fig. 4A). However, the proportion of NMD-sensitive transcripts among DNE1 targets (21%) was mostly similar to those found in nontargets and others (background sets, 17%). These results suggest that while some DNE1 targets are sensitive to NMD, the majority are not.

A well-known transcript feature that often promotes NMD is an uORF in the 5′ UTR (Nyikó et al. 2009; Rayson et al. 2012; Shaul 2015; Karousis et al. 2016). An uORF can initiate and terminate translation upstream of a protein-coding start codon; notably, the stop codon of an uORF can be recognized as premature and thus activate NMD. In the Arabidopsis genome, 84 transcripts have been annotated to contain an uORF (Araport 11; Cheng et al. 2017), and of those, 56 were detected in our seedling RNA degradome (CPM ≥ 1). We found 8 transcripts containing uORFs among DNE1 targets, and the observed frequency was significantly higher than nontargets and others (*P* < 0.001, as determined by the Fisher’s exact test; Fig. 4A).

NMD typically targets mRNAs with a PTC that may occur more than 50 to 55 nucleotides upstream of an exon–exon junction (Nagy and Maquat 1998; Nyikó et al. 2013). In human cell lines, a large subset of SMG6 cleavage sites were found near a termination codon (TC) within 50 nt of a downstream exon–exon junction (Schmidt et al. 2015). However, DNE1 cleavage sites do not seem to occur in the proximity of exon–exon junctions (Fig. 3C); therefore, we speculate that DNE1 functions independently of the EJC, which is different from the SMG6–EJC interaction known to occur in metazoans (Kashima et al. 2010). Our interpretation is supported by the fact that no known EJC factors were identified in the DNE1 interactome (Schiaffini et al. 2022). Another transcript feature that induces NMD is a long 3′ UTR. Increasing the physical distance (≥ 350 nt) between the stop codon and PABP at the poly(A) tail leads to less efficient translation termination and increases the susceptibility of the mRNA to NMD (Peccarelli and Kebaara 2014). About 44% of the DNE1 targets had 3′ UTR lengths ≥ 350 nt, and this was modestly significant (*P* < 0.01, as determined by the Fisher’s exact test) compared with nontargets (35%; Fig. 4A). Collectively, these results indicate that a subset of DNE1 targets have features that can induce NMD.

The RNA degradome provides a global view of uncapped/truncated transcripts by capturing a mix of ribosome-free and ribosome-associated decay intermediates as well as endoRNase cleavage products. XRN4 is a major facilitator of co-translational mRNA decay, and its absence leads to stabilization of RNA decay intermediates on polysomes (Yu et al. 2016; Carpentier et al. 2020). In a recent study that used seedlings, similar to our work, co-translational substrates of XRN4 were identified by comparing RNA decay intermediates (GMUCT) against mRNA enriched on polysomes identified by RNA-seq (Carpentier et al. 2020). We analyzed if DNE1 targets were among the co-translational substrates of XRN4. Around 19% of DNE1 targets (*N* = 40, *P* < 0.001; Fisher’s exact test; Fig. 4A) were overrepresented among the substrates of XRN4, indicating that these mRNAs are also targets of co-translational mRNA decay. One reason this small proportion of DNE1 targets is associated with co-translational decay substrates of XRN4 could be because the latter are primarily decapped. Whether or not DNE1 associates with polysomes or associates with DCP1 during co-translational decay remains to be established.

EndoRNases can accelerate the decay of specific mRNAs, and therefore, we hypothesized that DNE1 targets could have higher or lower stability compared with other mRNAs. We examined the published data from global RNA decay measurements of Arabidopsis mRNAs (Sorenson et al. 2018) to compare RNA half-lives (*t*_1/2_) between DNE1 targets and nontargets. Indeed, we found DNE1 targets to be far less stable (median RNA *t*_1/2_ ∼55 min) than nontargets (median RNA *t*_1/2_ ∼108 min; *P* < 2.2E−16, as determined by the Wilcoxon test; Fig. 4B). The proportion of unstable transcripts among DNE1 targets was significantly higher than among nontargets and others. For instance, almost 48% of DNE1 targets (102 out of 214 transcripts) were short-lived transcripts (RNA *t*_1/2_ ≤ 60 min) compared with nontargets (24%) and others (21%). The subset of DNE1 targets that overlapped with co-translational decay substrates showed a median RNA *t*_1/2_ ≤ 37 min (Supplemental Data Set S6), indicating that these transcripts undergo fast turnover on polysomes. NMD targets tend to have shorter RNA half-lives than other transcripts (Tani et al. 2012; Raxwal et al. 2020). Interestingly, less than 27% (27 out of 102 transcripts) of unstable DNE1 targets were NMD-sensitive, indicating that NMD is not the de facto reason for shorter half-lives of DNE1 targets. Taken together, these results indicate that RNA instability is a prominent feature of DNE1 targets.

To analyze if RNA instability of DNE1 targets is disrupted when 5′ to 3′ decay is blocked or impaired, we took advantage of RNA *t*_1/2_ studies in mutants of *VCS* (Sorenson et al. 2018) and *DDH1/DDX6-like* RNA helicases (Chantarachot et al. 2020). Compared with Col-0, the median RNA *t*_1/2_ of DNE1 targets was found to be 1.8× and 2.3× longer in *vcs*-7 and *rh6812* mutants, respectively (Fig. 4C and Supplemental Data Set S6). VCS and DDH1/DDX6-like RNA helicases are key factors of mRNA decapping, and their loss leads to widespread disruption of RNA half-lives across the Arabidopsis transcriptome (Sorenson et al. 2018; Chantarachot et al. 2020). As expected, our analysis indicates that DNE1 targets are also substrates of decapping-dependent mRNA turnover. In our bioinformatic analysis of DNE1 targets, we found 70 out of 224 transcripts with Major internal 5′P sites also accumulated highly abundant decapped intermediates (Supplemental Data Set S1). To analyze the effects of DNE1 on the abundance at the decapping sites, we compared the log_2_ fold-change distribution between *xrn4*/Col-0 and *dne1 xrn4*/Col-0 at XRN4-sensitive decapped sites. For this analysis, XRN4-sensitive 5′P sites (*N* = 2,522 from 1,418 transcripts) that were coincident with the decapping site (Cap-PARE, Nagarajan et al. 2019) were used. Interestingly, the median abundance at XRN4-sensitive decapping sites compared with Col-0 was significantly higher in *dne1 xrn4* than *xrn4* (Fig. 4D), supporting the idea that mRNA decapping serves as an alternative decay pathway not only for DNE1 targets but also for most degrading mRNAs when DNE1 is absent.

### 3′ RNA fragments of DNE1 targets overaccumulate in *xrn4*

**Figure 5.**
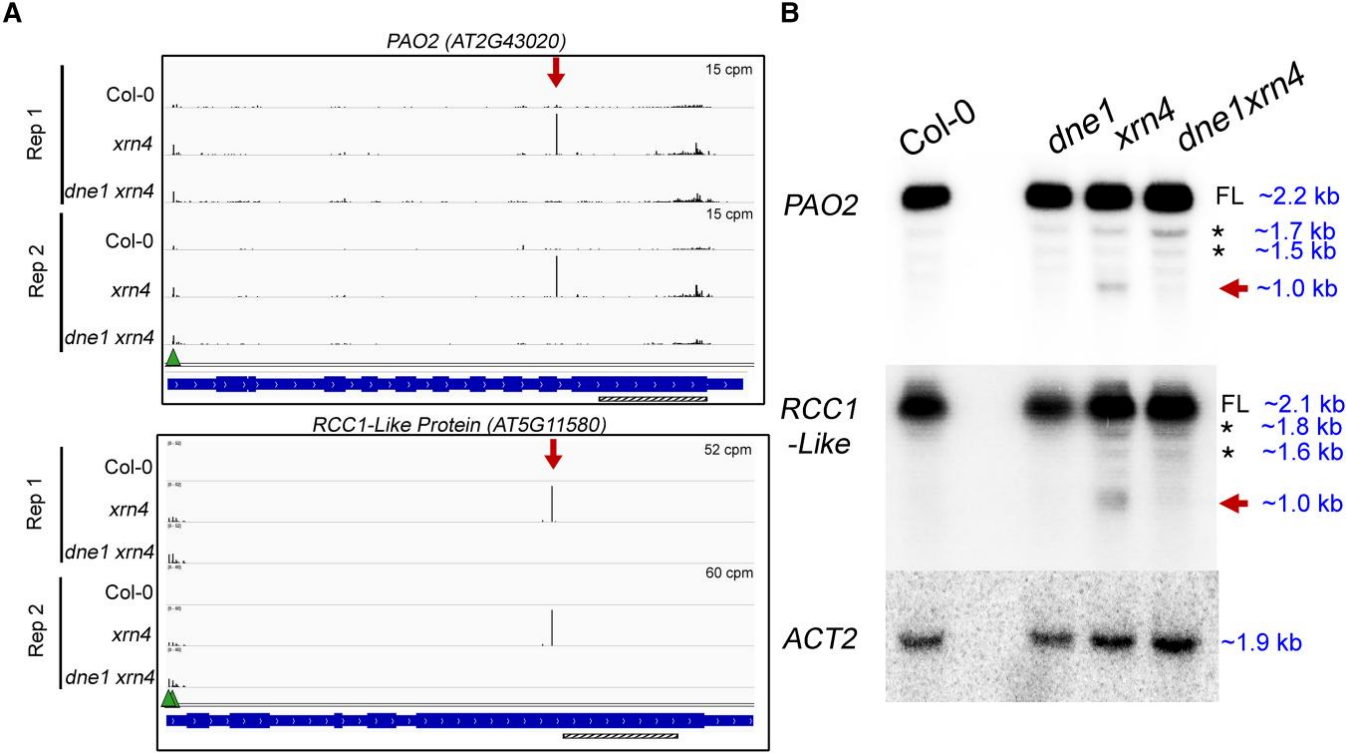
Examples of DNE1 cleavage sites detectable in *xrn4*. **A)** Decay (D) plots from GMUCT libraries showing increased abundances at DNE1 cleavage sites within *PAO2* and *RCC1-Like* transcripts in *xrn4* seedlings. Decay profiles for these transcripts from PARE libraries are shown in Supplemental Fig. S6. *Y* axis, CPM; arrow, MaxSeq; triangle, decapping site; gene structure from IGV shows introns and exons. Hatched bar, position of the 3′ probe used for RNA blots in **B**. **B)** RNA blot analysis of 3′ RNA fragments in *xrn4* seedlings that correspond to decay intermediates from the DNE1 cleavage sites in **A**. *ACT2* is shown as a loading control. Total RNA blots are representative of at least three biological replicates. Molecular weights of the hybridization bands were determined by comparing them with the 0.5 to10 kb RNA ladder (Life Technologies) that was loaded on the gel. FL, full-length transcript; asterisk, nonspecific bands; arrow, 3′ RNA fragment.

Based on the RNA degradome analysis, we examined the decay profiles of a few DNE1 targets in more detail. Two of these, *PAO2* and *RCC1-Like* transcripts, overaccumulate MaxSeqs within the CDS that are present in *xrn4* and absent in *dne1 xrn4* (Fig. 5A). Decay (D) plots, which help visualize the RNA degradome results on a per-transcript basis, show reproducibility of the MaxSeq within *PAO2* and *RCC1-Like* transcripts in 2 biological replicates (Fig. 5A). Consistent with these results, the RNA decay profiles for these transcripts from the leaf PARE libraries also showed the same MaxSeq in *xrn4* (Supplemental Fig. S6). Next, modified 5′ RNA Ligation-mediated Rapid Amplification of cDNA Ends (RLM-RACE) confirmed that the 5′ end of the major fragment overaccumulating in *xrn4* matches the MaxSeq positions detected within *PAO2* and *RCC1-Like* transcripts (Supplemental Fig. S6). Using additional biological replicates (3 to 5, Supplemental Table S1), we then tested whether the MaxSeqs that correspond to 3′ RNA fragments could be detected via RNA blot analysis. For both transcripts, a probe downstream of the MaxSeq position detected the full-length mRNAs (∼2.2 kb, *PAO2*; ∼2.1 kb, *RCC1-Like*) as well as short RNA fragments (∼1 kb) that corresponded to the prominent 3′ RNA fragments generated in the absence of XRN4 (Fig. 5B). Importantly, these short RNA fragments were not detectable in *dne1 xrn4* (Fig. 5B). In addition to the full-length and 3′ RNA fragments, in both *PAO2* and *RCC1-Like* transcripts, the 3′ probe also hybridized with at least 2 more additional bands that were mostly detectable in all genotypes. We used a 5′ probe upstream of the cleavage sites of *PAO2* and *RCC1-Like* transcripts to determine the nature of these nonspecific bands (Supplemental Fig. S7). The 5′ probe specifically detected 1 of the 2 bands (1.7 kb, *PAO2*; 1.8 kb, *RCC1-Like*) in Col-0, *xrn4*, and *dne1 xrn4*, indicating that these could be unannotated isoforms. These products are not 5′ RNA fragments arising from DNE1 cleavage, since they are not the expected size (∼1.1 kb for both *PAO2* and *RCC1-Like* mRNAs). It is known that 5′ RNA decay fragments are difficult to stabilize, and their fast turnover would explain why they are not detected in Col-0 and *xrn4*. This also indicates that the 3′ RNA fragment is detectable only downstream of the cleavage site. Overall, across multiple experiments and biological replicates, our results demonstrate that DNE1 is required to produce the 3′ RNA fragments of *PAO2* and *RCC1-Like* transcripts.

We also examined 2 additional transcripts, *HB-6* and *RAP2.4*, which show a DNE1-dependent MaxSeq and a Major internal site, respectively, in *xrn4*. The MaxSeq within *RAP2.4* coincides with the decapping site and is among the most abundant RNA decay intermediates that overaccumulate in *xrn4* and in *dne1 xrn4* (Supplemental Fig. S8A). However, a Major internal site within the 3′ region of the *RAP2.4* CDS overaccumulates in *xrn4* and is mostly absent in *dne1 xrn4* (Supplemental Fig. S8A). For both *HB-6* and *RAP2.4*, a 3′ probe downstream of the MaxSeq position detected the full-length mRNAs as well as short RNA fragments that corresponded to the prominent 3′ RNA fragments generated in the absence of XRN4 (Supplemental Fig. S8B). Of note, multiple 3′ RNA fragments (asterisk, Supplemental Fig. S8B) arising from *RAP2.4* were detected in *xrn4* that correspond to abundant DNE1-dependent 5′P sites that occur within 100 nt flanking the Major site. Together, these results raise the possibility that DNE1 could cleave at multiple sites within this transcript (Supplemental Fig. S8A) and others (Supplemental Data Sets S1 and S3). Unlike our previous analysis of endoRNase targets (Schmidt et al. 2015; Nagarajan et al. 2019), which identified one prominent site per transcript, our current analysis is more robust and allows for the detection of multiple 5′P sites with differential accumulation between *xrn4* and *dne1 xrn4*. This was especially useful in detecting highly abundant sites that are secondary to a prominent decapped intermediate, such as those found within the *RAP2.4* CDS.

### A conserved residue within the NYN domain of DNE1 is required for *PAO2 and RCC1-Like* 3′ cleavage

**Figure 6.**
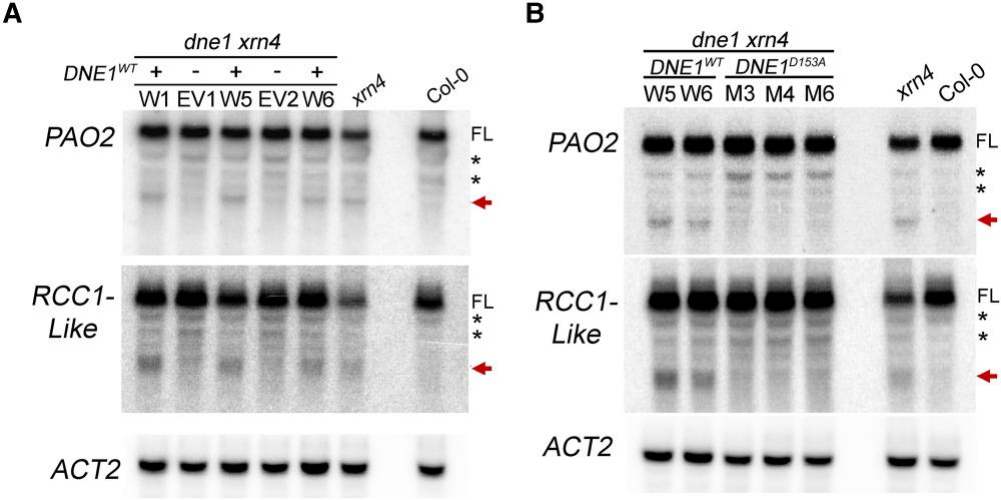
Key residue within the NYN domain of DNE1 is required for the production of 3′ RNA fragments. RNA blot analysis of 3′ RNA fragments of *PAO2* and *RCC1-Like* transcripts in two-week-old *dne1 xrn4* seedlings expressing different transgenic constructs. Independent T2 lines carrying empty vector (−) or overexpressing DNE1 (+) either as a WT or an active-site (D153A) mutant copy were assayed. **A)** RNA levels are shown for plants expressing either the CFP-tagged wild-type coding sequence of *DNE1* (*DNE1^WT^*, W) or the *35S:CFP* empty vector (EV). **B)** Transgenic plants overexpressing either the CFP-tagged wild-type coding sequence of *DNE1* (*DNE1^WT^*, W) or the corresponding D153A active-site mutant of *DNE1* (*DNE1^D153A^*, M). Total RNA blots are representative of two independent biological replicates. The position of the D153 residue is shown in Fig. 1B. Levels in Col-0 and *xrn4* are shown alongside. *ACT2* is shown as a loading control. Molecular weights of the bands were determined as per the legend of Fig. 5B. FL, full-length transcript; asterisk, nonspecific bands; arrow, 3′ RNA fragment.

To confirm that DNE1 is responsible for the generation of 3′ RNA fragments, *35S:CFP:DNE1* (*DNE1^WT^*) and the *35S:CFP* empty vector (EV) were introduced into *dne1 xrn4* double mutants (Supplemental Fig. S9). RNA blot analysis was performed using total RNA extracted from pooled seedlings of Col-0, *xrn4*, and independent T2 lines. The T2 lines expressing *DNE1^WT^* were selected based on their high expression of *DNE1*, much greater than that of Col-0 (Supplemental Fig. S9B). For the *PAO2* and *RCC1-Like* transcripts, 3′ probes detected the full-length mRNAs as well as the 3′ RNA fragments in *dne1 xrn4* stably expressing *DNE1^WT^* but not in *dne1 xrn4* expressing the EV (Fig. 6A). These results indicate that DNE1 is required to produce the 3′ RNA fragments of these mRNAs, which supports the role of DNE1 as a functional endoRNase. To confirm that these 3′ RNA fragments are generated by the endoRNase activity of DNE1, a mutant version of the enzyme with an inactive NYN domain was developed. Functional studies of metazoan MARF1 have indicated that D272 within the NYN domain is essential for the nuclease activity of the enzyme (Nishimura et al. 2018; Yao et al. 2018). The corresponding residue in Arabidopsis DNE1 is D153 (Fig. 1B), so we mutated this residue to alanine and introduced the *DNE1^D153A^* construct into *dne1 xrn4* double mutants. While Schiaffini et al. (2022) substituted an asparagine (N) residue for the aspartic acid (D) residue within the NYN domain, we used a neutral alanine (A) residue to eliminate the catalytic activity of the endoRNase, similar to the strategy used in the mouse MARF1 mutagenesis study (Yao et al. 2018). Transgenic T2 lines expressing *DNE1^WT^* and *DNE1^D153A^* were selected for further analysis based on comparable expression of *DNE1* (Supplemental Fig. S9B). While the 3′ RNA fragments of *PAO2* and *RCC1-Like* transcripts were easily detectable in *DNE1^WT^* lines (Fig. 6B), they were absent in *DNE1^D153A^* lines (Fig. 6B). These observations confirm that the NYN domain of DNE1 is required to cleave the targets identified in our study.

## Discussion

Our work captures endogenous RNA substrates of a MARF1 enzyme in a model organism and demonstrates the requirement of its NYN endoRNase domain for generating these cleavage products. MARF1-related Arabidopsis DNE1 is a recently discovered NYN domain–containing endoRNase that interacts with major mRNA decay factors, such as the NMD effector UPF1 and the decapping cofactor DCP1 (Chicois et al. 2018; Schiaffini et al. 2022). Therefore, it is both timely and important to understand the role of DNE1 in plants and identify its endogenous substrates. In our study, we identified targets of Arabidopsis DNE1 using a global approach that combines detection of its RNA substrates and their corresponding XRN4-sensitive 3′ decay intermediates. Our work provides new insights about the features of DNE1 targets and the potential involvement of this endoRNase in mRNA degradation pathways in Arabidopsis.

Transcriptome-wide mapping of cleavage sites is a thorough approach used to identify endoRNase substrates, but it has been performed for only a handful of endoRNases (e.g. Lykke-Andersen et al. 2014; Schmidt et al. 2015; Park et al. 2019; Hurtig et al. 2021). Our analysis indicates that DNE1 plays a role in the turnover of over 200 mRNAs in Arabidopsis seedlings. Most DNE1-dependent cleavage sites occur within exons (Figs. 3A and S5A), indicating that DNE1 could be involved in a cytoplasmic RNA quality control mechanism, in accordance with its localization in the cytosol and within P-bodies (Fig. 1, D and E; Chicois et al. 2018; Schiaffini et al. 2022). A major finding from our study is that DNE1 contributes to the turnover of a small subset of NMD-sensitive transcripts. In our previous work, we showed that 5′P sites corresponding to 3′ RNA fragments are enriched among NMD-sensitive transcripts, indicating that an unknown endoRNase may be involved in this decay pathway (Nagarajan et al. 2019). Due to its interaction with UPF1, plant DNE1 was an ideal candidate to perform this task, akin to SMG6 in metazoans. In our analysis, we found that 21% of DNE1 targets (46 out of 224 transcripts; Raxwal et al. 2020) are among those elevated in a *upf1* mutant (Fig. 4A). Some of these transcripts are known to contain uORFs within their 5′ UTR, a well-established NMD trigger. One such example is the *PAO2* transcript, which produces a DNE1-dependent 3′ RNA fragment; in the absence of DNE1, or when a catalytic residue within the NYN domain is mutated, this RNA fragment is no longer produced (Figs. 5 and 6). As expected, *PAO2* mRNA levels are elevated in a *UPF1* mutant allele *upf1-1* (Supplemental Fig. S10C) and appear to be increased in a *smg7* mutant (Gloggnitzer et al. 2014), indicating that it is an NMD target.

Another example is the *RCC1-Like* transcript, which also produces a DNE1-dependent 3′ RNA fragment (Figs. 5 and 6). Although the *RCC1-Like* transcript shows increased expression in *upf1* (Supplemental Fig. S10C), it has no known NMD triggers other than an annotated splice variant. It is possible that there are several unannotated splice variants for this gene, based on public data sets that capture full-length cDNA (TIF-seq, Thomas et al. 2020). Additionally, our data indicate that multiple decapping sites lie within the 5′ UTR of *RCC1-Like* (Figs. 5 and S6). Based on these observations, we can infer that this gene produces several splice variants and that some of them could be aberrant.

Our analysis indicates that most DNE1 targets are not NMD-sensitive and that DNE1 could have functions in general mRNA decay. This is consistent with the lack of UPF1-dependent changes to the expression of many DNE1 targets, including *RAP2.4* and *HB-6*. The *dne1* mutation does not reduce the accumulation of the 3′ RNA fragment from the known NMD target *eRF1-1* in *xrn4* (Nagarajan et al. 2019), indicating that DNE1 endoRNase activity is not required for this process (Supplemental Fig. S10, A and B). It is likely that this transcript and possibly other NMD-sensitive transcripts are cleaved by endoRNases that have yet to be identified. Given the complexity of plant NMD, it is very likely that several RNA decay pathways participate in the turnover of aberrant transcripts. Further work using different NMD mutants will be required to understand how DNE1 targets NMD-sensitive mRNAs. Overall, our work shows that DNE1 targets select uORF-containing, NMD-sensitive, and NMD-insensitive transcripts, indicating that this endoRNase is important for the turnover of a diverse set of mRNAs.

MARF1 and DNE1 proteins contain multiple repeats of the LOTUS domain, which has been recently shown to bind G-rich and G-quadruplex sequences (Ding et al. 2020). In our study, the G-quadruplex prediction programs could not identify such structures within DNE1 target sequences (Supplemental Table S4). Interestingly, a short G-rich motif (YGGWG; Fig. 3, E and F) was highly enriched within a 10-nt region flanking the cleavage site of DNE1 targets. The majority of DNE1 targets, including those we examined in more detail (*PAO2*, *RCC1-Like*, *RAP2.4*, and *HB-6*), showed 1 or more occurrences of YGGWG within a 20-nt region flanking the cleavage site, indicating that this is a common feature of most DNE1 targets. The presence of such G-rich RNA sequences can form complex structures and folding states, resulting in high thermostability due to the unusual base stacking confirmation. For these RNAs to be translated or degraded, RNA helicases must unfold the complex structures. While the RNA helicase activity of UPF1 is important for NMD, it is also required for other RNA decay pathways (Kim and Maquat 2019). Metazoan UPF1 interacts with certain endoRNases that cleave structured regions on mRNAs so that the helicase activity of UPF1 can promote subsequent mRNA degradation (Mino et al. 2015; Fischer et al. 2020). Therefore, it is possible that the interaction between UPF1 and DNE1 and subsequent RNA decay is unrelated to NMD. Several RNA helicases, including UPF1, were among the top candidates that co-precipitated with DNE1 (Schiaffini et al. 2022), so it would be interesting to see if DNE1-mediated mRNA decay is dependent on the helicase activity of UPF1 and/or other RNA helicases.

An intriguing observation from our bioinformatic analysis to identify DNE1 targets was that a third of these transcripts also accumulated abundant decapped intermediates. Thus, DNE1 targets are also substrates of the decapping-dependent mRNA turnover pathway. Decapping is a major route for mRNA turnover in Arabidopsis, and its impacts are apparent from the severe seedling lethal phenotypes observed for decapping mutants (Xu et al. 2006; Goeres et al. 2007; Iwasaki et al. 2007). We did not evaluate the contributions of deadenylation that precedes decapping, but due to the relationship between DNE1 and DCP1, we predict that these 2 proteins and their respective decay pathways may compete for substrates. Therefore, we should expect a concomitant and additive increase in the abundance of decapped intermediates when DNE1 and 5′ to 3′ decay (e.g. XRN4) are simultaneously blocked. In the absence of DNE1, there was an increased abundance at decapping sites for XRN4-sensitive transcripts (Fig. 4D), implying that DNE1 could influence 5′ to 3′ decay. The finding that there is a significant overlap between transcripts with increased expression in the *vcs-7* mutant (Sorenson et al. 2018) and the DNE1 overexpression (OE) line (Schiaffini et al. 2022) provides genetic evidence for the interaction between decapping- and DNE1-mediated mRNA decay. A scenario suggested by Schiaffini et al. (2022) is that DNE1 OE might compromise the formation of the decapping complex because DNE1 competes for DCP1, resulting in the overlap between transcripts increased in the DNE1 OE line and the *vcs* mutant. This implies that most transcripts with increased abundance in the DNE1 OE line could be decapping substrates rather than cleaved DNE1 substrates, which would explain why we observed very few (9) transcripts with both increased abundance in the DNE1 D153N OE line and DNE1-dependent cleavage sites. Nevertheless, our results examining XRN4-sensitive decay intermediates confirm that decapping- and DNE1-mediated mRNA decay share many RNA substrates, with the former pathway being dominant. This is a possible reason why DNE1 is not sufficient to rescue the seedling lethal phenotype of decapping mutants.

A major finding of our work was that DNE1 targets are intrinsically unstable. A large proportion of endogenous transcripts that undergo DNE1-mediated decay are short-lived [median RNA *t*_1/2_ = 55 min (Fig. 4B) and 68 min (Fig. 4C)], from 2 different studies. These RNA half-lives were at least 2-fold shorter than what was observed for nontargets. This is noteworthy considering XRN4-sensitive decapped targets have a median RNA *t*_1/2_ of >80 min for polyadenylated transcripts (Nagarajan et al. 2019). Overall, our result provides support for 2 major inferences about DNE1 targets: (i) they are difficult to detect due to their prominent instability and (ii) they are eliminated by parallel or redundant RNA decay pathways. For example, DNE1 targets *RAP2.4* and *HB-6* are well-known examples of unstable mRNAs. The former is targeted by a sequence-specific mRNA decay mechanism (Perez-Amador et al. 2001), and the latter was identified as one of the Genes with Unstable Transcripts (AtGUTs, Gutiérrez et al. 2002). Both transcripts also accumulate abundant decapped intermediates, indicating that they utilize an alternate RNA decay pathway. Likewise, in mammalian cells, even though the highly unstable inflammatory and cytokine mRNAs (such as TNF-a and interleukin-6) preferably undergo endoRNase-mediated decay, they will also be degraded by alternative decay pathways if necessary (Boehm et al. 2016). Our investigation of DNE1-mediated mRNA decay supports the view that other mechanisms of RNA regulation involving endoRNases have yet to be discovered (Li et al. 2010; Tomecki and Dziembowski 2010).

**Figure 7.**
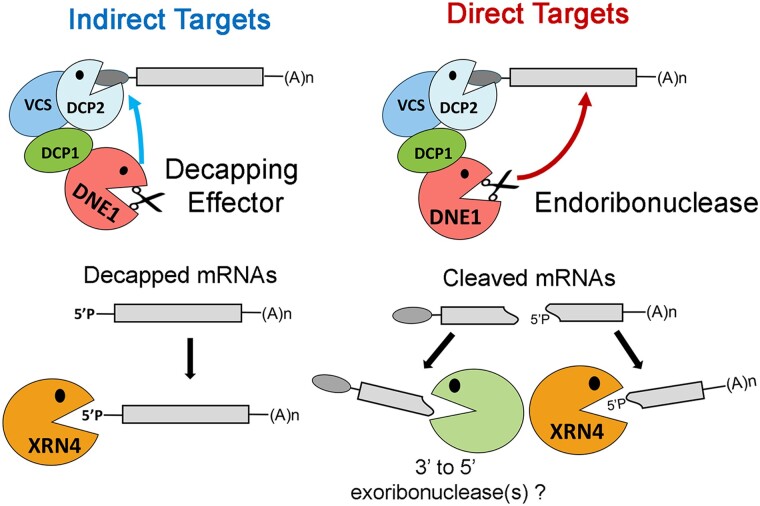
Model showing the dual role of DNE1 in cytoplasmic mRNA decay. DNE1 can act as a decapping effector promoting mRNA decapping and 5′ to 3′ decay of mRNAs in an indirect manner. Direct targets are cleaved by the endoribonuclease action of DNE1, and the resulting cleaved RNA fragments are degraded by XRN4 (5′ to 3′ decay) and other exoribonucleases (3′ to 5′ decay). Whether or not DNE1 needs to be associated with DCP1 to cleave direct mRNA targets is unknown.

Our work provides substantial information on the transcripts and sequences targeted by DNE1. We also identified novel associations between DNE1 and products of abiotic stress-responsive genes (e.g. those involved in ABA signaling) as well as developmental genes involved in organ morphogenesis (Supplemental Table S3). Given the shorter mRNA half-lives for most of the DNE1 targets, posttranscriptional control appears to be important for the expression of these genes. DNE1 may target mRNAs directly and indirectly, and this dual role would make it more effective at controlling gene expression at the level of mRNA stability. If mRNA stability is disrupted for even a small number of transcripts, it could have a bearing on gene expression and lead to biological defects in response to various regulatory stimuli, such as those reported for mutants with DNE1 dysfunction (Luhua et al. 2008, 2013; Schiaffini et al. 2022). We posit a model (Fig. 7) wherein DNE1, by its interaction with DCP1, promotes mRNA decapping followed by 5′ to 3′ decay of mRNAs (indirect targets). Additionally, the endoRNase activity of DNE1 results in rapid decay of mRNAs (direct targets) in a sequence-specific manner (e.g. G-rich sequences) that may require additional co-factors (e.g. RNA helicases). It has been proposed that DNE1 could be part of a subcomplex with DCP1 within the cell (Schiaffini et al. 2022); however, it is not known if this association is required for DNE1 target recognition and/or mRNA cleavage.

In conclusion, we have demonstrated that Arabidopsis DNE1 is a functional endoRNase with a direct role in mRNA decay. This study globally identifies endogenous cleaved targets of a MARF1 family member in a eukaryotic organism (Arabidopsis). Our study also lays the groundwork for understanding the molecular mechanism(s) by which DNE1 participates in plant mRNA decay. It seems likely that DNE1, potentially in connection with UPF1 and/or DCP1, offers plants a mechanism to accelerate mRNA decay. This would facilitate rapid responses to stimuli such as environmental stresses, something that is particularly valuable to sessile organisms. The overrepresented categories of transcripts among DNE1 cleavage targets should provide clues to identify new biological impacts of the enzyme in plants, a strategy that proved fruitful previously in the study of XRN4 in transgenic Arabidopsis (Nagarajan et al. 2019).

## Materials and methods

### Plant materials and growth conditions

Arabidopsis (*Arabidopsis thaliana*) ecotype Col-0, *xrn4* (*xrn4-5*, Souret et al. 2004), *dne1* (*dne1-1*, SALK_132521), and *upf1* (*lba1*, Yoine et al. 2006) in the Col-0 background were used for these studies. The *dne1 xrn4* double mutants were generated by reciprocal crossing of single homozygous mutants (*xrn4* and *dne1*) and identified from segregating F2 populations via genotyping PCR. Seeds were surface-sterilized with 70% (*v*/*v*) ethanol followed by 3.25% (*v*/*v*) sodium hypochlorite and stratified in the dark at 4 °C for 48 h. Seeds were plated on germination media containing 1× Murashige and Skoog (MS) salts, 1× B5 vitamin mix, 1.0% (*w*/*v*) sucrose, 0.5% (*w*/*v*) 2-(*N*-morpholino)-ethanesulfonic acid (MES), and 1% (*w*/*v*) Phyto agar, adjusted to pH 5.7 (Nagarajan et al. 2019). Surface-sterilized seeds from transgenic lines were grown on selection medium containing 0.5× MS salts, 1.0% (*w*/*v*) sucrose, 0.5% (*w*/*v*) MES, and 0.7% or 1% (*w*/*v*) Phyto agar supplemented with appropriate antibiotics. Seedlings were allowed to germinate under constant light (Philips ALTO II) for 7 h and then transferred to dark for 48 h to improve selection as described before (Harrison et al. 2006). Seedlings where then moved to long-day conditions of 16 h light/8 h dark at 21 °C and grown normally prior to harvesting for RNA or soil transfer. Parental lines were similarly handled and grown alongside in selection media without the antibiotics. Plants were cultivated in soil (Pro-Mix) under climate-controlled long-day conditions at 21/22 °C, with weekly water and fertilizer (Miracle-Gro plant food) regimes and grown to maturity.

### Plasmid construction and generation of transgenic lines

The coding region of *DNE1* (1.47 kb from ATG to TAA) was PCR amplified from cDNA isolated from Col-0 plants using primers that added *BsrG*I and *Spe*I sites to the 5′ and 3′ ends, respectively. The purified PCR product was A-tailed with GoTaq DNA polymerase and subcloned into pGEM-T Easy (Promega, Durham, NC, USA) to generate p2619. Subsequently, the *DNE1* ORF was released from the p2619 by restriction digest using *BsrG*I and *Spe*I (New England Biolabs, Ipswich, MA) and ligated to the *35S:CFP* empty vector (p2622) that had been cut with the same enzymes to generate *35S:CFP:DNE1* (*DNE1^WT^*, p2624). To generate the NYN domain active-site mutation, the 5′ and 3′ regions of the *DNE1* coding region were separately PCR amplified using primers that carried the D153A mutation and added *PspOM*I and *Spe*I sites to the 5′ and 3′ ends, respectively. Since the primers consisted of regions that overlapped between the 2 regions of *DNE1*, the gel-purified PCR products were fused together using the Gibson Assembly method (New England Biolabs, Ipswich, MA) according to the manufacturer's instructions. The purified PCR product was subcloned into pGEM-T Easy (p2673) as before. Subsequently, the mutant *DNE1* ORF was released from p2673 by restriction digest using *PspOM*I and *Spe*I (New England Biolabs, Ipswich, MA) and ligated to p2624 that had been cut with the same enzymes to generate *DNE1^D153A^* (p2676).

All final constructs were verified by Sanger sequencing and mobilized into *A. tumefaciens* (GV3101 pMP90) electrocompetent cells. Agrobacterium-mediated plant transformations were carried out using the floral dip method (Clough and Bent 1998). All PCR amplifications were carried out using Phusion High-Fidelity DNA Polymerase (Thermo Fisher Scientific, Waltham, MA, USA).

### DNE1 domain architecture and multiple sequence alignment

The domain architecture of Arabidopsis DNE1 was determined using the SMART algorithm (http://smart.embl-heidelberg.de/). A multiple sequence alignment of the NYN domains of DNE1 (NP_179158.2), HsMARF1 (NP_001171927.1), and MmMARF1 (NP_001074623.1) was performed using the BLOSUM62 algorithm within Multalin (default parameters) (Corpet 1988). The residues required for ssRNase activity as determined by Yao et al. (2018) were subsequently annotated within this alignment.

### Subcellular localization

Fluorescent fusion proteins were transiently expressed in fully expanded *N. benthamiana* leaves (Caplan et al. 2015) or stably expressed in transgenic Arabidopsis plants. Subcellular localizations were imaged in *N. benthamiana* leaves 3 d after Agrobacterium-mediated infiltration using a LSM880 confocal microscope (Carl Zeiss NTS Ltd.) with a 40× water immersion objective (NA = 1.2) using a laser excitation wavelength of 458 nm. Root epidermal cells of 7-d-old Arabidopsis T2 lines were imaged using the high-speed Dragonfly spinning disk confocal microscope (Andor Technology Oxford Instruments) with a 40× water immersion objective (NA = 1.1) using a laser excitation wavelength of 445 nm. All images were processed using ImageJ or Imaris Viewer.

### Total RNA extraction and cDNA synthesis

Total RNA was extracted using TRI reagent (Molecular Resource Center, Inc., Cincinnati, OH, USA) according to the manufacturer's instructions, with the addition of an acid phenol/chloroform cleanup step at the end. Total RNA (2 *μ*g) was DNase-treated prior to oligo(dT) priming and was reverse transcribed using SuperScript II (Thermo Fisher Scientific, Waltham, MA, USA).

RT-PCR was performed on Applied Biosystems MiniAmp Thermal Cycler (Thermo Fisher Scientific, Waltham, MA, USA) using the GoTaq Master Mix (Promega, Durham, NC, USA) and 0.4 *μ*M of primer. Housekeeping gene *ACT2* (Czechowski et al. 2005) was PCR amplified to test for uniformity of cDNA synthesis.

### Quantitative RT-PCR

Gene expression was analyzed using RT-qPCR on a Bio-Rad CFX96 Optics Module. Each RT-qPCR reaction contained cDNA diluted 1:10, 2× Premix Ex Taq SYBR Green (Takara Bio, San Jose, CA, USA), and 0.5 *μ*M of each primer. Pooled seedlings from at least 3 biological replicates or as indicated per genotype per experiment were sampled, and each reaction was run in triplicate. Relative levels were computed by the 2^−ΔΔCt^ method of quantification (Livak and Schmittgen, 2001) and normalized to *ACT2*.

### RNA blots

Total RNA (15 to 20 *μ*g) was resolved on MOPS-formaldehyde denaturing gels solidified with 1.5% or 1.8% (*w*/*v*) agarose and blotted onto Hybond N+membranes. DNA probes were PCR amplified from Col-0 cDNA using the primers listed in Supplemental Table S2. The purified PCR products were radiolabeled using the Thermo Scientific DecaLabel DNA Labeling Kit according to the manufacturer's instructions. Membranes were hybridized in PerfectHyb Plus Hybridization Buffer (Sigma-Aldrich, St. Louis, MO, USA) with the 32P-dCTP-labeled DNA probes at 65 °C overnight. All membranes were washed with 2× SSC, 0.1% SDS for 10 min at 65 °C, followed by 1 or 2 washes of 0.2× SSC, 0.1× SDS for 10 min at 65 °C. Signal intensities were analyzed using the Typhoon system (GE Health Sciences). Membranes were stripped in boiling 0.1% SDS for 20 min between hybridizations. All northern blot results presented are representative of 2 or more biological experiments.

### 5′ RACE

A modified RNA ligase-mediated (RLM) 5′ RACE was performed using the FirstChoice RLM-RACE kit (Thermo Fisher Scientific, Waltham, MA, USA) as described in Nagarajan et al. (2019). Briefly, DNase-treated total RNA (10 *μ*g) was ligated to a 5′ RNA adapter (GUUCAGAGUUCUACAGUCCGAC) and reverse transcribed with oligo(dT). The cDNA was first amplified by a 30- or 35-cycle PCR, and the resulting dsDNA was further amplified by a 30-cycle PCR using internal primers.

All primers used in the study are listed in Supplemental Table S2.

### RNA-seq library construction and analysis

Poly(A)^+^RNA was fractionated from seedling total RNA (10 *μ*g) using a standard RNA magnetic bead-based oligo(dT) purification (Poly(A) Purist MAG Kit, Thermo Fisher Scientific, Waltham, MA, USA). RNA-seq libraries were generated using the NEBNext Ultra II RNA Library Prep Kit (New England Biolabs) following the manufacturer's instructions. Size distribution and concentration of the libraries were estimated using the Fragment Analyzer Automated CE System (Agilent Technologies, Inc.). Libraries were multiplexed and sequenced as 75 nt single-end reads on Illumina NextSeq 550. RNA-seq reads were checked for quality using FastQC v0.11.9 (http://www.bioinformatics.babraham.ac.uk/projects/fastqc/). The reads were mapped to the Arabidopsis TAIR10 reference genome using TopHat v2.1.1 (Trapnell et al. 2009) with the following parameters: *library type*, *fr-firststrand*; *transcript features and junction coordinates from the TAIR10 GTF*. Finally, transcript abundance levels were compared to determine differential expression using Cufflinks/CuffDiff v2.2.1 (Trapnell et al. 2012). The criteria for differentially accumulating protein-coding transcripts and noncoding RNA were as follows: RPKM ≥ 2 in mutant or Col-0; FC: ≥ 1.5 in either direction (log_2_ FC ≥ 0.58); and false discovery rate (FDR)-adjusted *P* ≤ 0.05. Three biological replicates of each library were used in the overall analysis.

### RNA degradome library construction

Poly(A)^+^ RNA isolated from 6-week-old rosette leaves or 2-week-old whole seedlings were used for generation of PARE and GMUCT libraries, respectively.

PARE: Steps to generate these libraries are described previously (German et al. 2009; Nagarajan et al. 2019). Libraries were sequenced on Illumina HiSeq 2500 in a 50-nt single-end mode.

GMUCT: Steps to generate these libraries are described previously (Carpentier et al., 2021) with the following changes. RNA was ligated to a 5′ RNA adapter containing an *EcoP15*I recognition site at its 3′ end (Li et al. 2019). The cDNA was purified using AMPure XP beads prior to final library amplification. Libraries were sequenced on Illumina NextSeq 550 in a 75-nt single-end mode.

### RNA degradome computational analysis

The fastq files from the current study were first checked for sequence quality using FastQC and then trimmed using Trimmomatic (PARE: CROP 20 and MINLEN 20; GMUCT: HEADCROP 5, CROP 50, and MINLEN 50). The additional HEADCROP parameter for GMUCT raw reads is to remove the extra 5 nt of the *EcoP15*I recognition site on the RNA adapter. The subsequent analysis was performed as described in Hurtig et al. (2021) using a command line version. Briefly, TopHat v2.0 was used to map fastq files to TAIR10 v49 genome. Using Bamcoverage (deepTools suite; Ramírez et al. 2016), the resulting bam files were normalized to counts per million (CPM), bin size = 1, and offset = 1 (only first nt of each read is counted), and positive and negative strands in the genome were analyzed separately to generate bedgraphs (PARE scores) and bigwig output files. The log_2_ FCs were computed from the bigwig files using Bigwigcompare (deepTools suite) with the following options: SkipZeroOverZero and Pseudocount 0.01 (to avoid division by 0 at each position) to generate a bedgraph file (comPARE scores). Finally, the bedgraph files were merged using unionbedg (Bedtools suite) to generate a file with both PARE scores (from Bamcoverage) and comPARE scores (from Bigwigcompare). Positions with low abundance scores in *xrn4* (PARE < 1 CPM) were removed from the merged bedgraph output files. The bedgraph output files were next annotated using (i) annotatePeaks (HOMER suite; Heinz et al. 2010) and (ii) intersect (Bedtools suite; Quinlan and Hall 2010), to identify sites across the genome and within genes, respectively. Getfasta (Bedtools suite) was used to extract sequences based on genomic coordinates. Additional filters: (i) Decapping site—Cap-PARE (C-PARE) libraries generated from our previous study (GSE119706, Nagarajan et al. 2019) were analyzed using the pipeline described above. These 5′P sites with an abundance ≥5 CPM were identified as mRNA decapping sites. This criterion was used to ensure that decapping sites were not misidentified as cleavage sites so positions near (±5 nt) the annotated TSS or experimentally verified decapping site were not considered. In our analysis, MaxSeq and Major internal sites dependent on DNE1 were not coincident with the decapping sites or annotated TSS (TAIR10 v49). (ii) Sequences that matched the genome and transcriptome more than once were also removed from the analysis. (iii) Transcript abundance from RNA-seq data was used to ensure that reduced abundance at a 5′P site in *dne1 xrn4* was not due to lower expressivity of the full-length target mRNA compared with *xrn4* or Col-0 (Supplemental Data Set S4). Cleavage and decapping sites identified from our analysis were manually checked using the Integrated Genome Viewer (IGV) visualization browser (Robinson et al. 2011).

Custom Perl and R scripts were used for further analysis of transcript features and can be accessed at https://github.com/nagvin/endoRNAse_NSF2018. Metagene analysis was conducted essentially as described by Olarerin-George and Jaffrey (2017), without the re-scaling feature for CDS and UTRs. Significance of overlap between data sets was tested using 2 methods—hypergeometric analysis and GeneSect analysis (a Monte Carlo based probability method in VirtualPlant version 1.3; Katari et al. 2010; Krouk et al. 2010). GO analysis of overrepresented biological processes was carried out using Biomaps (VirtualPlant version 1.3).

Motif discovery and enrichment analysis were performed using Multiple Em for Motif Elicitation (MEME) Suite 5.5.0 (Bailey and Elkan 1994). First, MEME was used to identify short motifs (5 to 7 nt) from 40 nt RNA sequences either spanning DNE1 cleavage site (Supplemental Data Set S1) or other internal sites that were XRN4-sensitive (i.e. *xrn4*/Col-0, log_2_ FC ≥ 1; *N* = 1,186) using the Differential Enrichment mode (with hypergeometric distribution function). Second, the degenerate motif with the lowest *E*-value (<0.005) identified within the DNE1 target sequence, YGGWG, was then verified using simple enrichment analysis (SEA). The DNE1 target sequences and nontarget sequences (described in the legend of Fig. 3) were compared for the enrichment of YGGWG and scored for true and false positive occurrences (FDR < 0.05). The sequences and transcripts from DNE1 targets and nontarget data sets used in the analysis in this study are mutually exclusive.

### Accession numbers

Raw and processed sequences of PARE, GMUCT, and RNA-seq libraries (Supplemental Table S1) are available at the National Center for Biotechnology Information (NCBI)-Gene Expression Omnibus (GEO) under the accession number GSE193247. Sequence data from this article can be found in the Arabidopsis Genome Initiative (https://www.arabidopsis.org/) under the following accession numbers: *ACT2/ACTIN 2*, *AT3G18780*; *DNE1*, *AT2G15560*; *HB-6*, *AT2G22430*; *PAO2*, *AT2G43020*; *RAP2.4*, *AT1G78080*; *RCC1-Like*, *AT5G11580*; *UBQ10*, *AT4G05320*; and *XRN4*, *AT1G54490*.

## Supplementary Material

koad085_Supplementary_Data

## Data Availability

The author responsible for distribution of materials integral to the findings presented in this article in accordance with the policy described in the Instructions for Authors (https://academic.oup.com/plcell/pages/General-Instructions) is: Pamela J. Green (greenpj@udel.edu).

## Supplemental data

The following materials are available in the online version of this article.

**Supplemental Figure S1.** Expression of DNE1 in *dne1* mutants.

**Supplemental Figure S2.** Correlation of 5′P site abundances between replicates of GMUCT and PARE libraries.

**Supplemental Figure S3.** Full-length and 5′P site abundance fold-change distribution of DNE1 targets.

**Supplemental Figure S4.** Computational pipeline for identifying DNE1 cleavage sites from rosette leaf PARE libraries.

**Supplemental Figure S5.** Features of DNE1 cleavage sites in leaf PARE analysis.

**Supplemental Figure S6.** DNE1 cleavage sites in *PAO2* and *RCC1-Like* identified from rosette leaf PARE libraries and their validation.

**Supplemental Figure S7.** Detection of the RNA decay intermediates of DNE1 targets using a 5′ probe.

**Supplemental Figure S8.** Additional prominent DNE1 cleavage sites identified from seedling GMUCT libraries and their detection.

**Supplemental Figure S9.** Expression of *DNE1* in transgenic lines.

**Supplemental Figure S10.** Relationship between UPF1 and DNE1.

**Supplemental Table S1.** Statistics of RNA-seq and RNA degradome libraries.

**Supplemental Table S2.** Primers used in the study.

**Supplemental Table S3.** Overrepresented GO terms among DNE1 targets.

**Supplemental Table S4.** G-quadruplex prediction within DNE1 target sequences.

**Supplemental Data Set S1.** DNE1 cleavage sites identified in seedlings using GMUCT.

**Supplemental Data Set S2.** Decapping sites identified from Cap-PARE analysis.

**Supplemental Data Set S3.** DNE1 cleavage sites identified in rosette leaves using PARE.

**Supplemental Data Set S4.** Transcripts up- and downregulated in *dne1* and *dne1 xrn4*.

**Supplemental Data Set S5.** Short sequence motif identified among DNE1 target sequences.

**Supplemental Data Set S6.** RNA half-lives of DNE1 targets.
