# Correction: RNA degradome analysis reveals DNE1 endoribonuclease is required for the turnover of diverse mRNA substrates in Arabidopsis

**DOI:** 10.1093/plcell/koad156

**Published:** 2023-05-31

This is a correction to: Vinay K Nagarajan and others, RNA degradome analysis reveals DNE1 endoribonuclease is required for the turnover of diverse mRNA substrates in Arabidopsis, The Plant Cell, 2023; koad085, https://doi.org/10.1093/plcell/koad085

In the originally published version of this manuscript, there was an error in Funding details. The first sentence of these should read: “This material is based upon work supported by the National Science Foundation under Grant No. MCB1817764 to P.J.G.” instead of: “This work was supported by the National Science Foundation (NSF-MCB1817764) to P.J.G.”.

This error has been corrected in the article.

