# Supplementary material for: RNA degradome analysis reveals DNE1 endoribonuclease is required for the turnover of diverse mRNA substrates in Arabidopsis
Source: Plant Cell. 2023 Apr 18;35(6):1936–55. doi: 10.1093/plcell/koad085 (PMC10226599; doi:10.1093/plcell/koad085)
Supplement: koad085_Supplementary_Data [file koad085_supplementary_data.zip › TPC2022RA00859R2_TPC2022RA00859R2_Supplemental_Figures_Tables_Final.docx]

**Supplemental Data**

**RNA degradome analysis reveals DNE1 endoribonuclease is required for the turnover of diverse mRNA substrates in Arabidopsis**

Vinay K. Nagarajan, Catherine J. Stuart, Anna T. DiBattista, Monica Accerbi, Jeffrey L. Caplan and Pamela J. Green


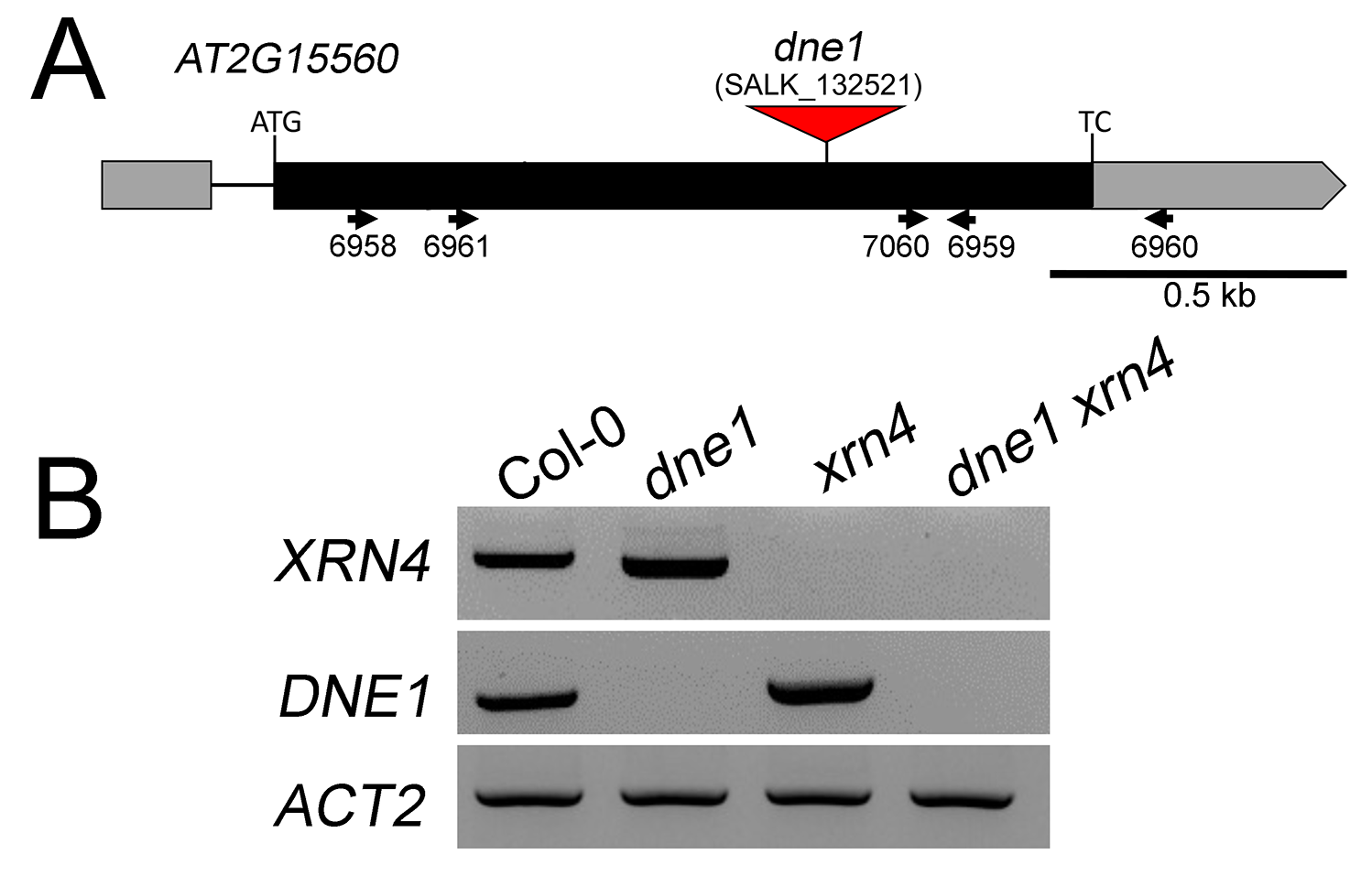


**Supplemental Figure 1**. **Expression of *DNE1* in *dne1* mutants (Supports Figure 2).**

**A)** Transcribed region of *DNE1* gene showing untranslated regions (gray), intron (black line), coding region (black), start and termination codons (ATG and TC, respectively). The T-DNA insertion line designated as *dne1* is indicated by the red triangle. Black arrows, primers used for genotyping (6960 and 6961), RT-PCR analysis in B (6958 and 6959), and Quantitative RT-PCR analysis (7060 and 6959) in Supplemental Figure 9.

**B)** RT-PCR results showing levels of *DNE1* and *XRN4* in homozygous single (*dne1* and *xrn4*) and double (*dne1 xrn4*) mutants. The *xrn4* allele is *xrn4-5* of Souret et al., 2004 and the *dne1* allele is *dne1-1* of Schiaffini et al., 2022. Total RNA for cDNA synthesis was isolated from rosette leaves pooled from several individual Col-0 or homozygous mutant plants. Gel images are representative of three biological replicates.

**
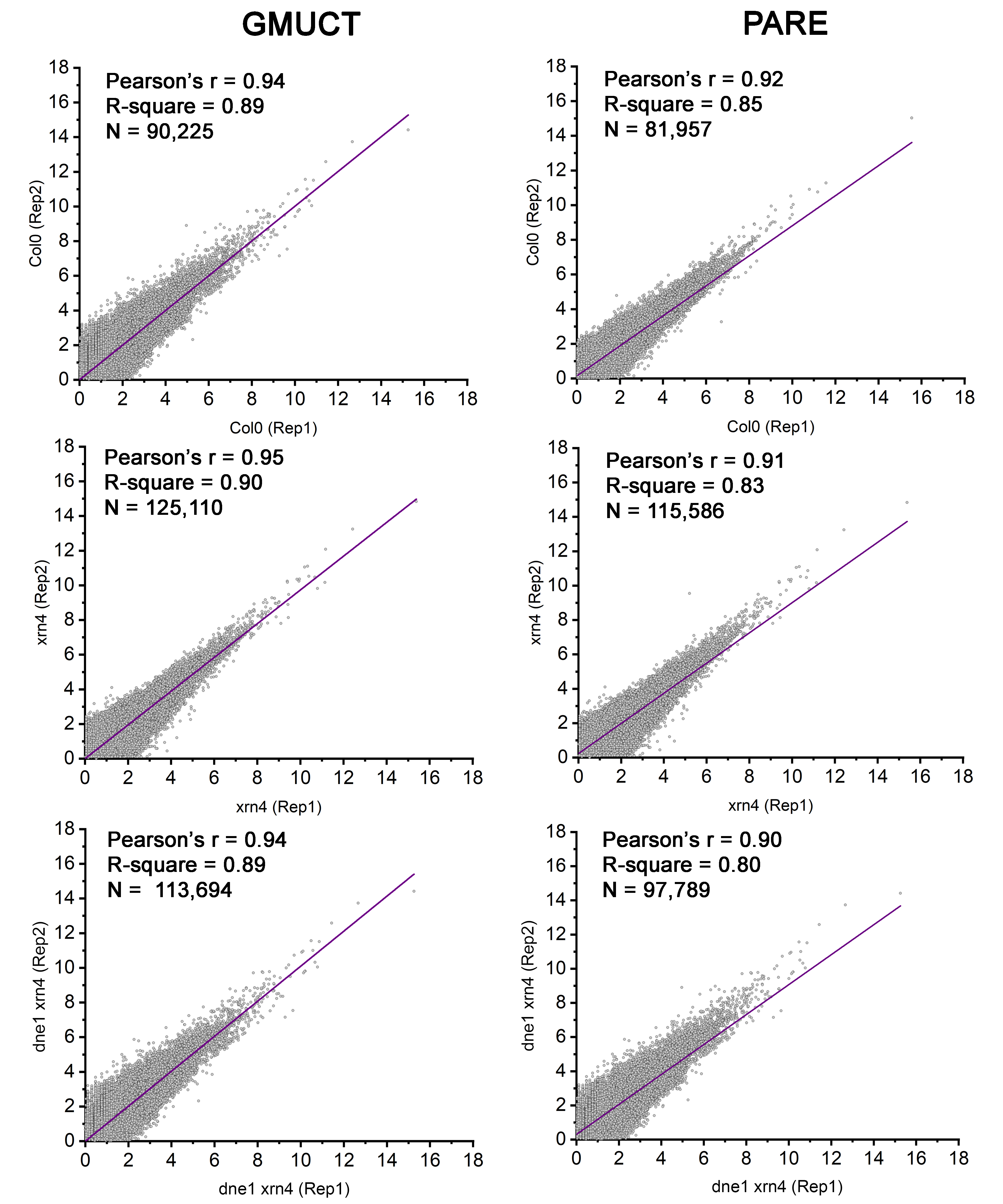
**

**Supplemental Figure 2. Correlation of 5’P site abundances between replicates of GMUCT and PARE libraries (Supports Figure 2).**

Scatter plots of log2 transformed 5’P site abundances (CPM > 1) between replicates of Col-0, *xrn4* and *dne1 xrn4*. Correlation coefficient (r) and R-square values are shown.

**
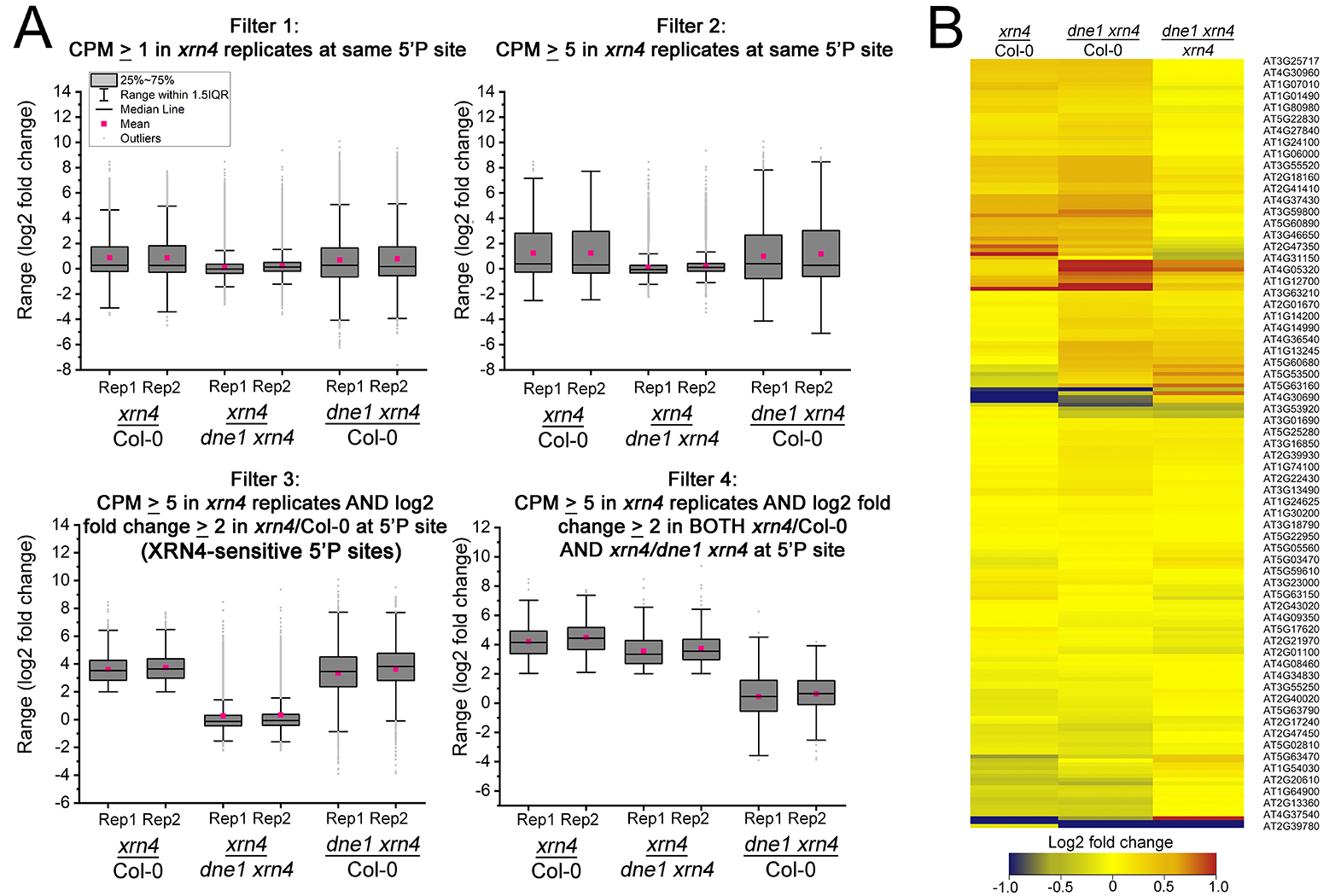
**

**Supplemental Figure 3. Full-length and 5’P site abundance fold-change distribution of DNE1 target transcripts (Supports Figure 2).**

**A)** Fold-change distribution for filters used in identifying XRN4-sensitive 5’P sites and DNE1-dependent 5’P sites from GMUCT libraries. Box plots of log2 transformed fold-changes of 5’P site abundances at major steps of the computational pipeline to identify DNE1 cleavage sites are shown. Refer to Figure 2 for more details of these filters and their output. The number of 5’P sites used in the analysis are as follows: Filter 1 (112,605), Filter 2 (15,260), Filter 3 (N = 4,738) and Filter 4 (N = 501). **B)** Heatmap of RNA-seq data shows full-length fold-changes of DNE1 target transcripts. Fold-changes (log2) of DNE1 target transcript (identified as per Figure 2) in different comparisons: *xrn4*/Col-0, *dne1 xrn4*/Col-0 and *dne1 xrn4*/*xrn4*. RNA-seq results are in Supplemental Data Sets S1 and S4.


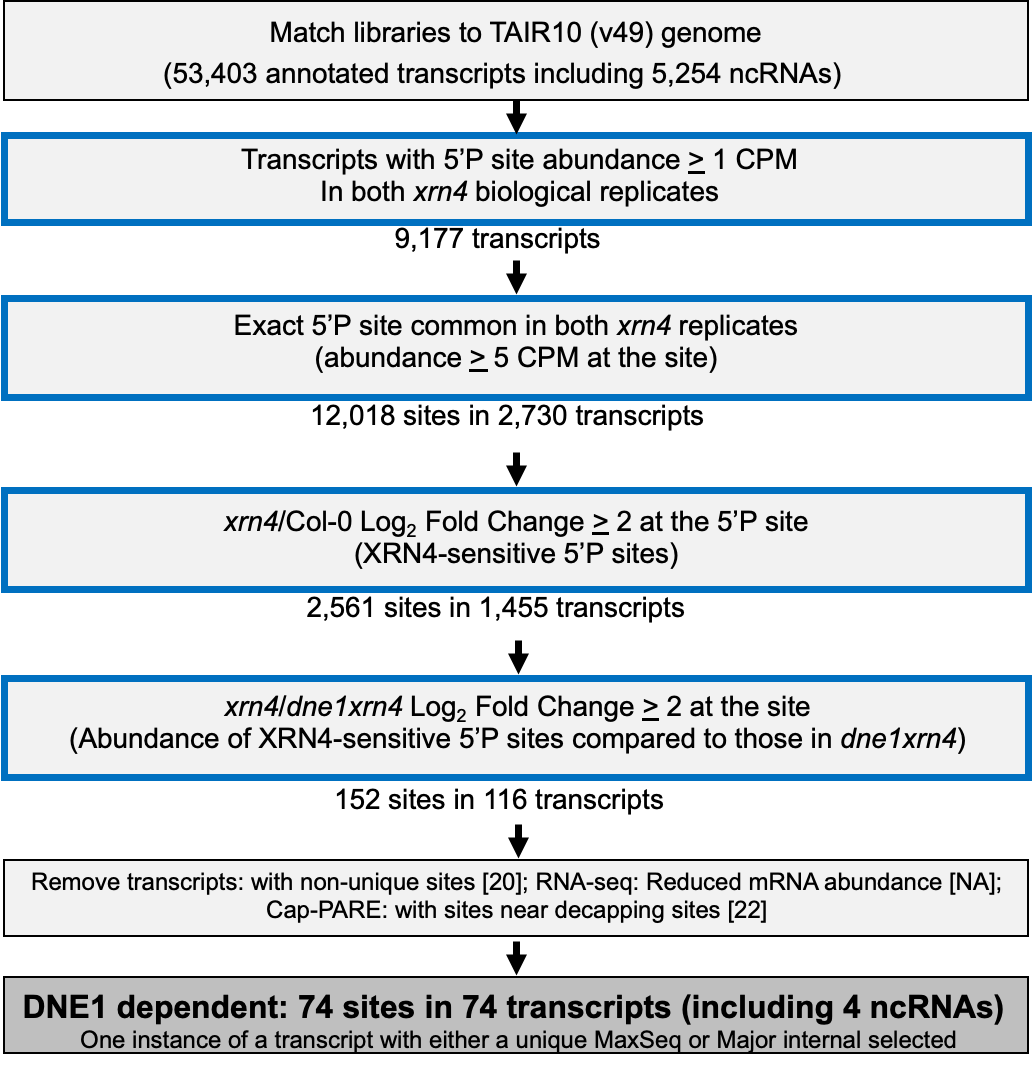


**Supplemental Figure 4. Computational pipeline for identifying DNE1 cleavage sites from rosette leaf PARE libraries (Supports Figure 2)**.

Output is highlighted in dark gray. Cleavage site corresponds to the position of the 5’P terminus of either a DNE1-dependent MaxSeq or a Major internal on a transcript. MaxSeq is the most abundant sequence on a transcript. Major internal is the next most abundant sequence when the most abundant sequence on a transcript is at the decapping site. The data are filtered (blue rectangles) using the criteria described in the Methods. Numbers in [ ] indicate transcripts that did not pass the filter. The transcript abundance filter was not used here since no RNA-seq results were available for the leaf samples. NA, not applicable.


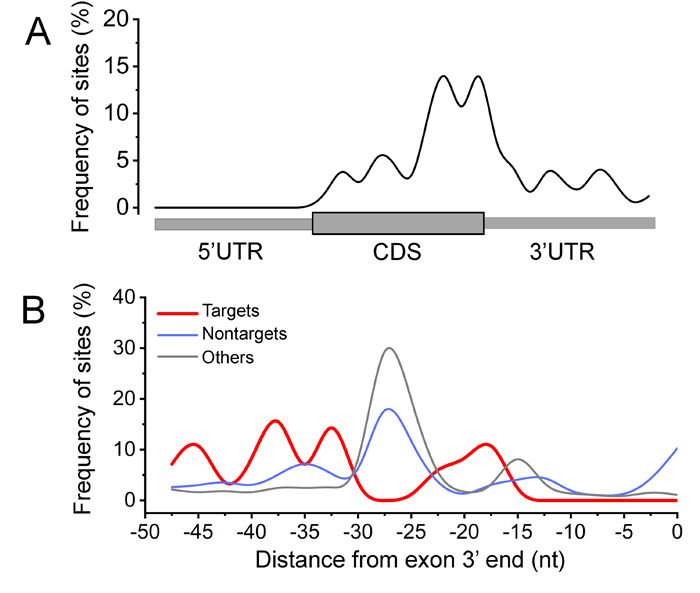


**Supplemental Figure 5. Features of DNE1 cleavage sites in leaf PARE analysis (Supports Figure 3).**

**A)** Metagene analysis of DNE1 cleavage sites. Analysis was performed as per the legend to Figure 3B. **B)** DNE1 cleavage sites are not enriched near exon-exon junctions. Distribution of relative frequency of MaxSeq and Major internal site occurrences in 50 nt region upstream of an exon-exon junction. Datasets, Targets (N = 48 sites in CDS), Nontargets (N = 702) and Others (N = 3,457) are described in the legend to Figure 3C.

**
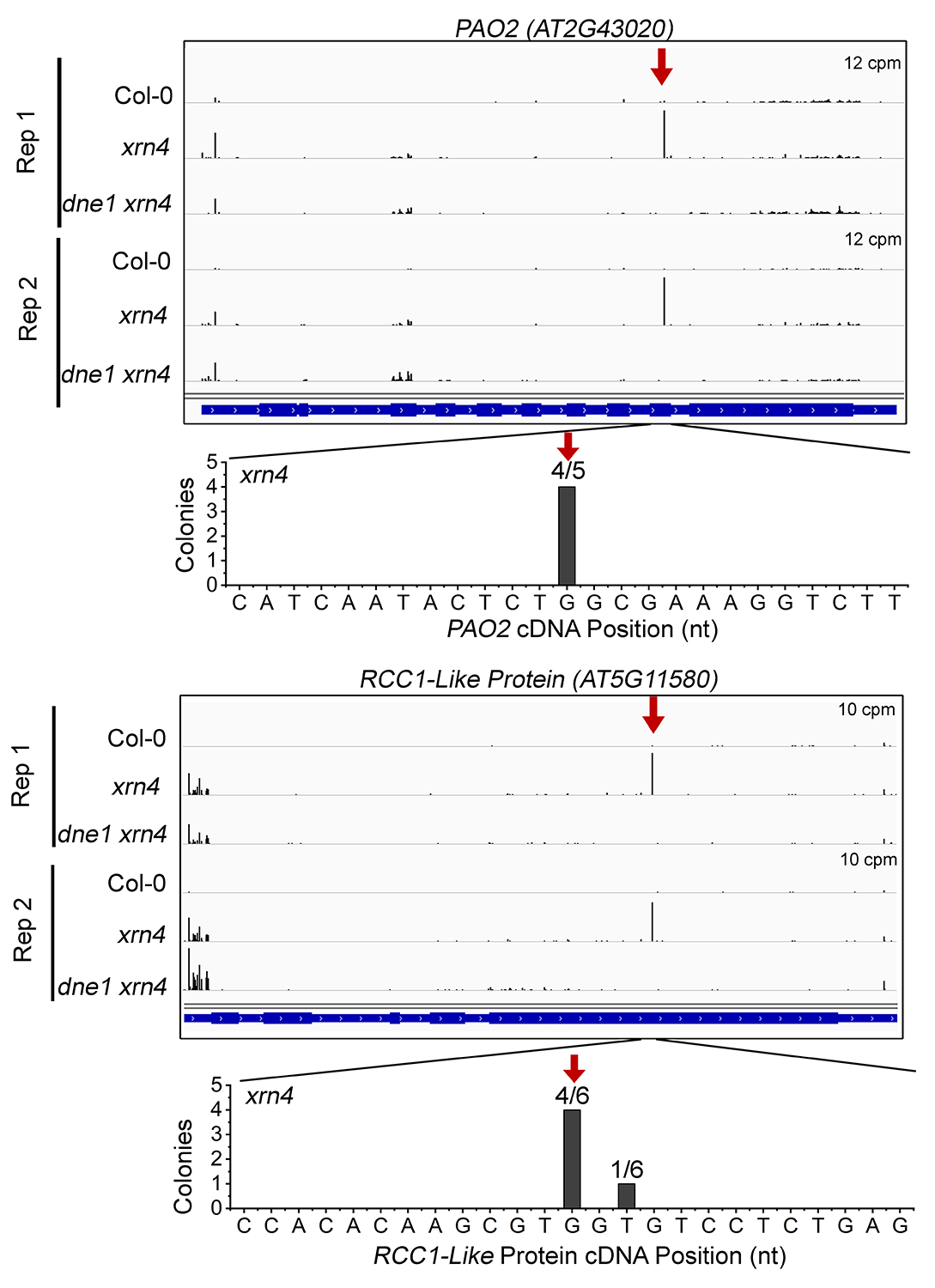
**

**Supplemental Figure 6. DNE1 cleavage sites in *PAO2* and *RCC1-Like* identified from rosette leaf PARE libraries and their validation (Supports Figure 5).**

D-plots are as per the legend to Figure 5A. Cleavage sites were verified by modified 5’RLM-RACE, and the results are shown below each plot. The number of cloned RACE products sequenced is shown above the bars. Arrow, MaxSeq.

**
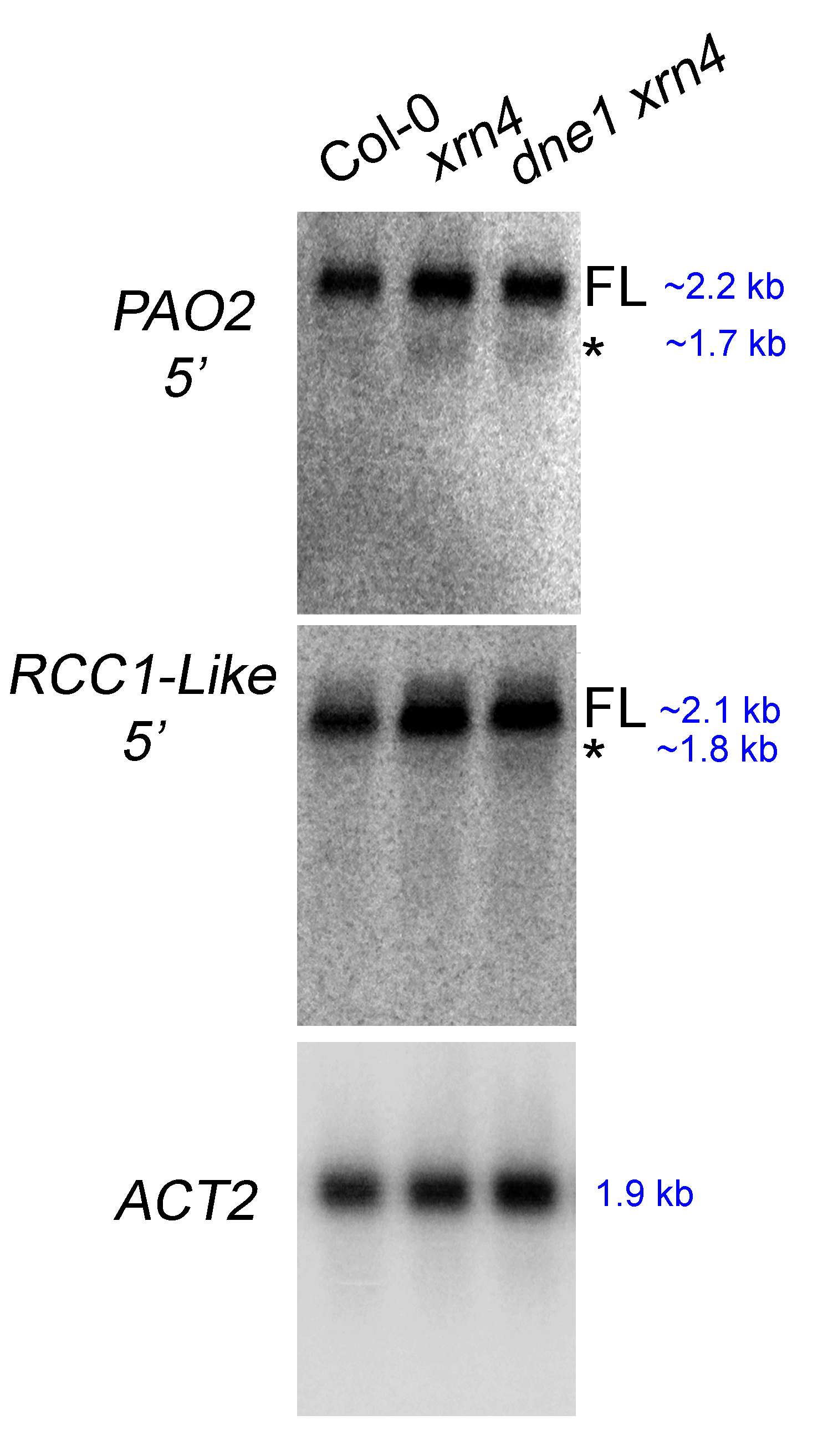
**

**Supplemental Figure 7. Detection of the RNA decay intermediates of DNE1 targets using a 5’ probe (Supports Figure 5).**

RNA blot analysis of *PAO2* and *RCC1-Like* transcripts in two-week-old Col-0, *xrn4* and *dne1 xrn4* seedlings using 5’ probes located at positions 268 nt and 162 nt upstream of the cleavage sites for *PAO2* and *RCC1-Like* cDNAs, respectively. FL, full-length transcript; Asterisk, non-specific bands. *ACT2* is shown as a loading control. Molecular weights of the bands were determined as per the legend to Figure 5B.


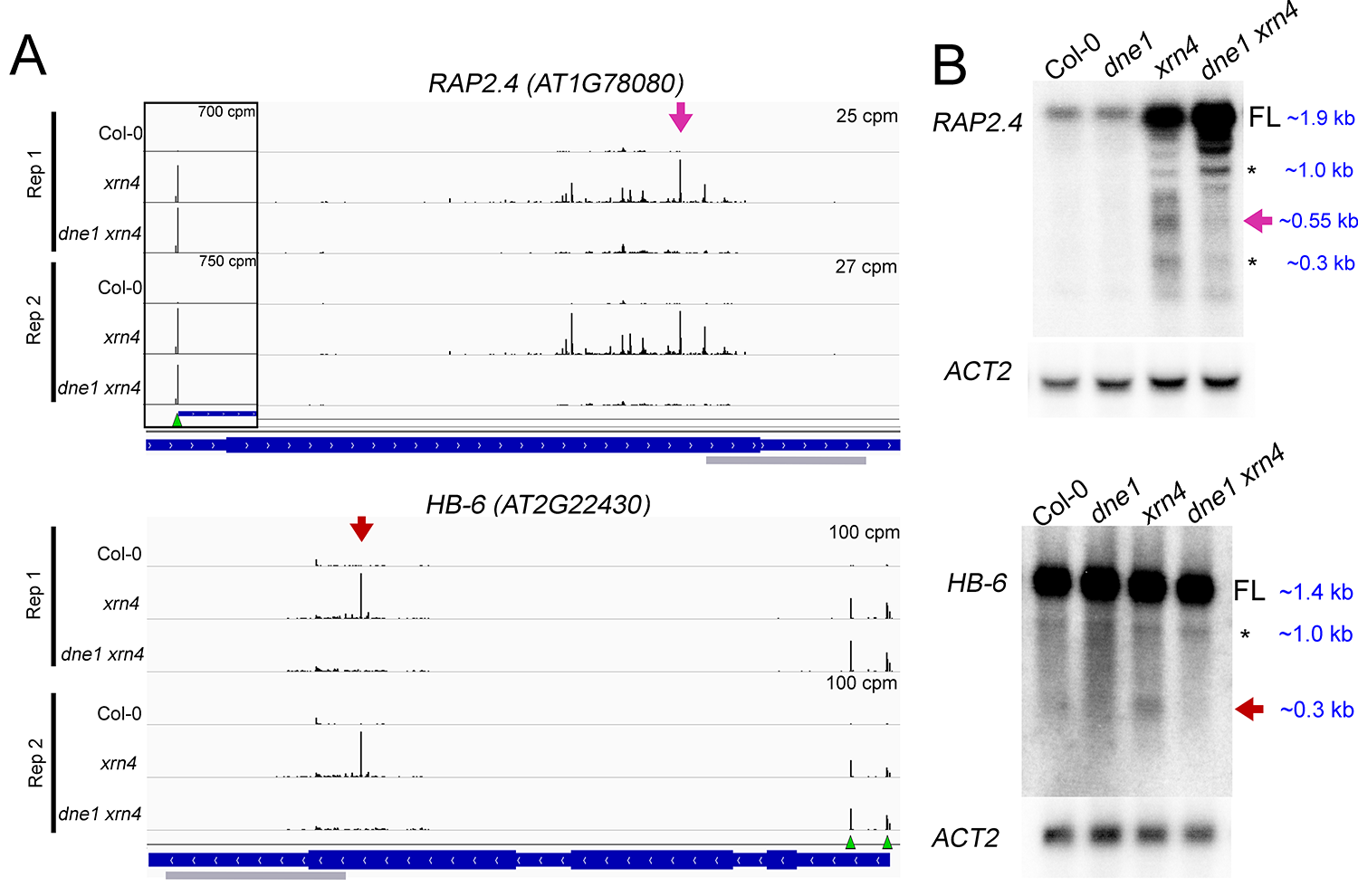


**Supplemental Figure 8. Additional prominent DNE1 cleavage sites identified from seedling GMUCT libraries and their detection (Supports Figure 5).**

**A)** D-plots from GMUCT libraries showing increased abundance at DNE1 cleavage sites within *RAP2.4* and *HB-6* transcripts in *xrn4* seedlings. Y-axis, CPM. Green triangle, decapping site; Pink arrow in *RAP2.4*, major internal site secondary to the decapping site. Inset, highly abundant *RAP2.4* decay intermediate at the decapping site in *xrn4* and *dne1 xrn4*. Red arrow in *HB-6*, MaxSeq; Gray bar, position of the 3’ probe used for RNA blots in B. **B)** RNA blot analysis of 3’ RNA fragments in *xrn4* seedlings that correspond to decay intermediates from the DNE1 cleavage sites in A. Molecular weights of the bands were determined as described in the legend to Figure 5B. FL, full-length transcript; Asterisk, non-specific bands; Arrow, 3’ RNA fragment. *ACT2* is shown as a loading control*.* Total RNA blots are representative of at least two biological replicates.


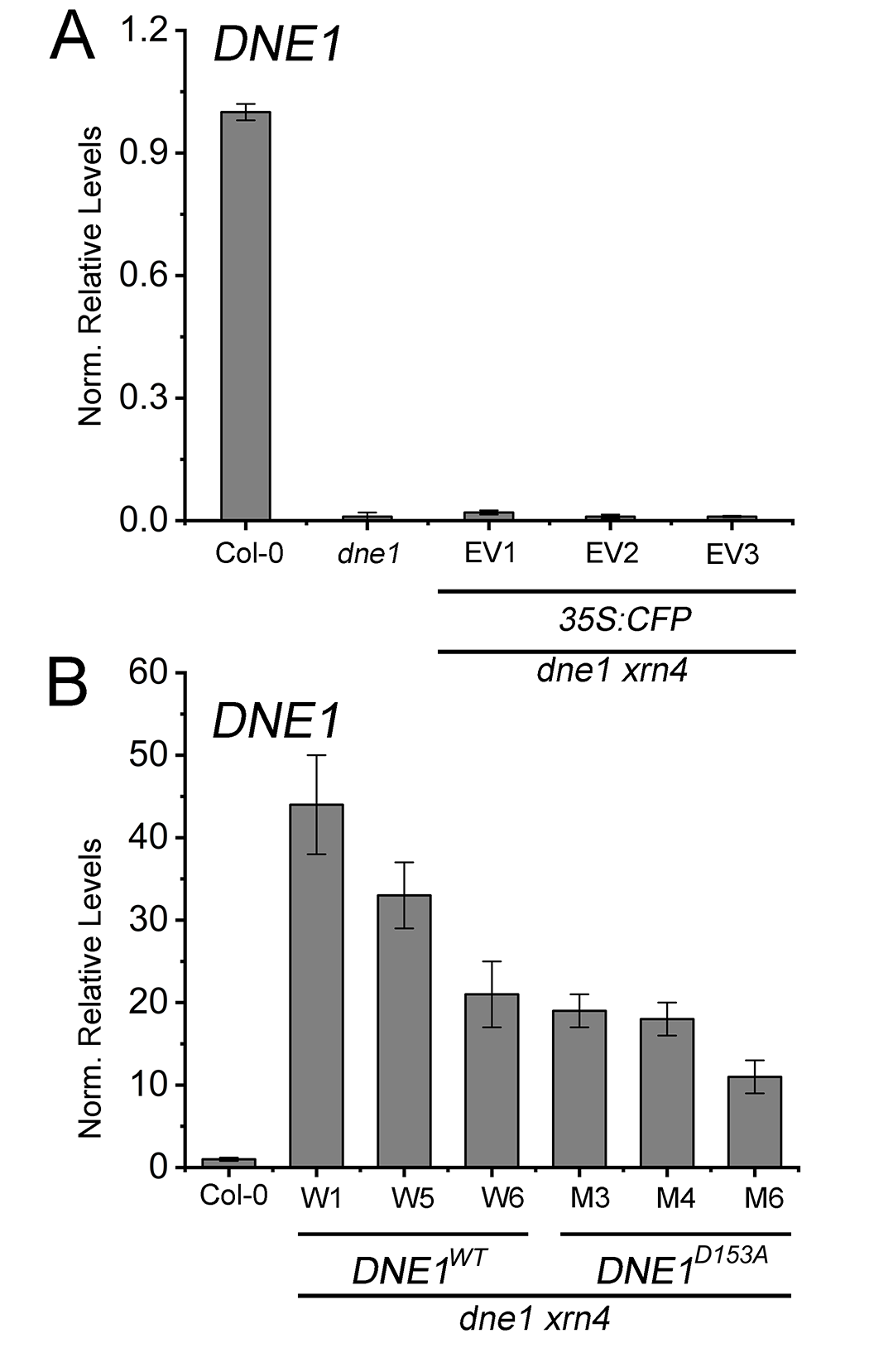


**Supplemental Figure 9. Expression of *DNE1* in transgenic lines (Supports Figure 6).**

Quantitative RT-PCR analysis of *DNE1* expression in *dne1 xrn4* plants (A) overexpressing the *35S:CFP* empty vector (EV), and (B) overexpressing the CFP-tagged wild-type coding sequence of *DNE1* (*DNE1^WT^,* W) or the corresponding D153A active-site mutant of *DNE1 (DNE1^D153A^,* M*)*. Relative *DNE1* levels normalized to *ACT2* from independent T2 lines expressing each construct are shown. Levels in Col-0 (set to 1) and *dne1* are shown alongside. Expression values are from two-week-old seedlings. Histograms are means + SD from two (A) and three (B) independent biological replicates.


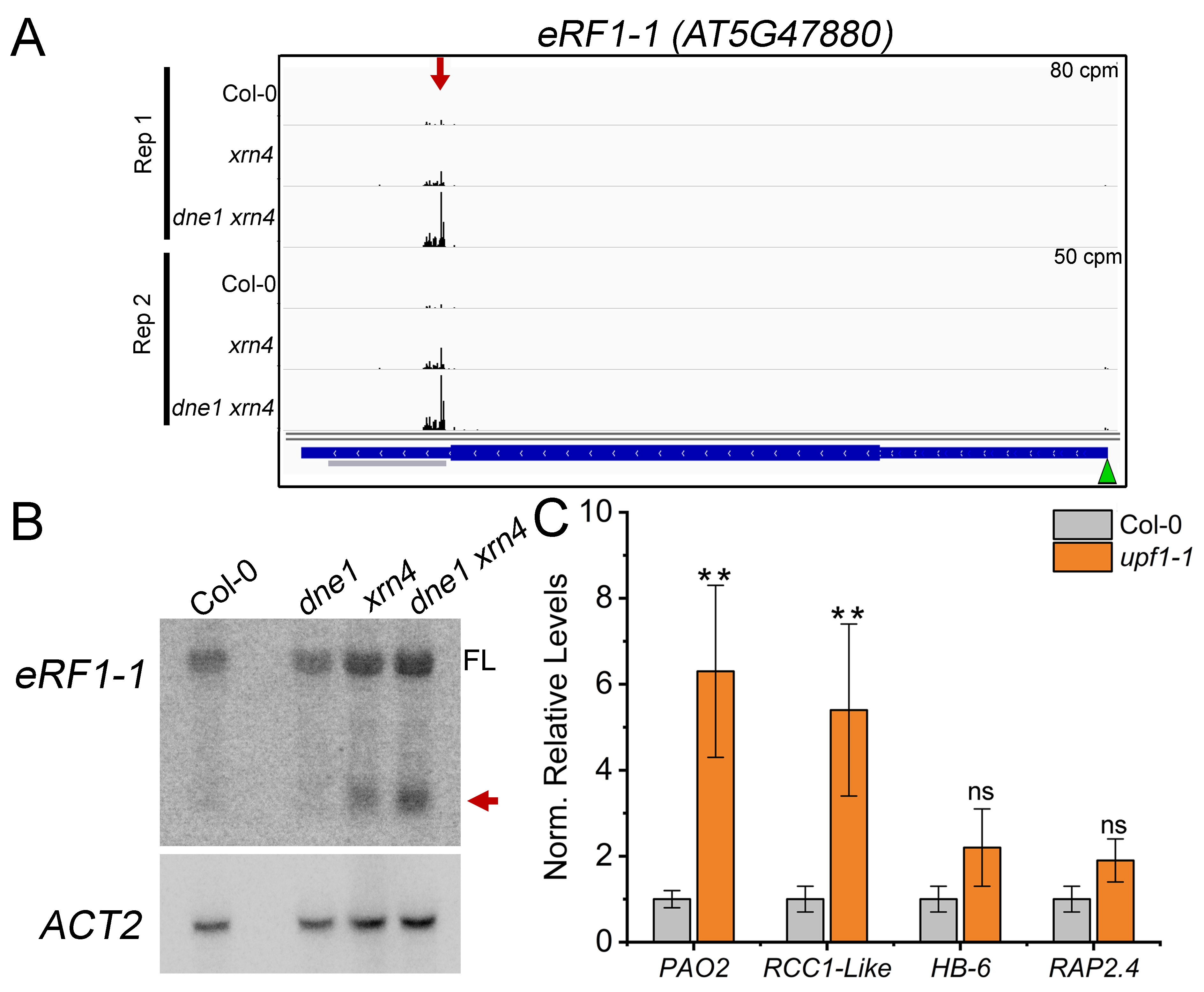


**Supplemental Figure 10. Relationship between UPF1 and DNE1 (Supports Figure 4).**

**A)** Accumulation of 3’ RNA fragment from NMD target *eRF1-1* in *dne1 xrn4* seedlings. D-plots from GMUCT libraries showing abundance at the site corresponding to the *eRF1-1* 3’ RNA fragment. Y-axis, CPM. Arrow, MaxSeq; Gray bar, position of 3’ probe used for RNA blots in B. Green triangle, decapping site. **B)** RNA blot analysis of 3’ RNA fragment from *eRF1-1* in *dne1 xrn4* seedlings. FL, full-length; Arrow, 3’ RNA fragment. *ACT2* is shown as a loading control*.* Total RNA blots are representative of at least two biological replicates. **C)** UPF1 dysfunction impacts expression of a few DNE1 targets. Quantitative RT-PCR analysis of DNE1-target transcripts in Col-0 and *upf1-1* seedlings. Relative levels of four transcripts were normalized to those of *ACT2.* Histograms are means + SD from three independent biological replicates. Levels in Col-0 were set to 1. P-values (**, P<0.001; ns, P>0.1) were determined by the Mann-Whitney U nonparametric test.

**Supplemental Tables**

**Supplemental Table 1. Statistics of RNA-seq and RNA degradome libraries.** Raw and processed RNA degradome and RNA-seq files are available on NCBI GEO under accession number GSE193247. Data processing details and analysis are described in the Supplemental Methods section.

|  | **RNA degradome Libraries** | | | | | | |
| --- | --- | --- | --- | --- | --- | --- | --- |
|  |  |  |  |  | Genome match (TAIR10) | | |
|  | Sample | Library Name | Raw Reads | Trimmed Reads | Distinct | Abundance | % Trimmed |
| **GMUCT** | Col-0 Seedling Rep 1 | ATH1082 | 31,481,634 | 30,051,856 | 9,146,971 | 28,491,497 | 94.8 |
|  | *xrn4* Seedling Rep 1 | ATH1083 | 22,056,548 | 20,956,801 | 7,530,138 | 20,025,811 | 95.6 |
|  | *dne1 xrn4* Seedling Rep 1 | ATH1084 | 15,958,877 | 15,385,610 | 5,488,393 | 14,921,004 | 97.0 |
|  | Col-0 Seedling Rep 2 | ATH1085 | 30,165,731 | 28,464,053 | 8,606,313 | 26,846,412 | 94.3 |
|  | *xrn4* Seedling Rep 2 | ATH1086 | 17,525,432 | 17,411,640 | 5,108,440 | 16,840,385 | 96.7 |
|  | *dne1 xrn4* Seedling Rep 2 | ATH1087 | 25,201,874 | 23,968,804 | 7,395,364 | 22,888,699 | 95.5 |
| **PARE** | Col-0 Leaf Rep 1 | ATH1036 | 47,986,364 | 44,987,171 | 7,024,961 | 36,988,283 | 82.2 |
|  | *xrn4* Leaf Rep 1 | ATH1037 | 48,820,758 | 45,727,004 | 8,178,517 | 42,799,925 | 93.6 |
|  | *dne1 xrn4* Leaf Rep 1 | ATH1038 | 58,298,315 | 54,893,694 | 8,668,155 | 49,468,422 | 90.1 |
|  | Col-0 Leaf Rep 2 | ATH1039 | 36,047,543 | 32,684,603 | 8,354,456 | 27,789,976 | 85.0 |
|  | *xrn4* Leaf Rep 2 | ATH1040 | 45,029,172 | 40,996,342 | 9,943,388 | 34,677,629 | 84.6 |
|  | *dne1 xrn4* Leaf Rep 2 | ATH1041 | 46,732,520 | 42,517,410 | 10,316,574 | 37,863,256 | 89.1 |

| **RNA-seq Libraries** | | | | |
| --- | --- | --- | --- | --- |
| Sample | Library Name | Raw Reads | TAIR10 Genome Matched Reads | % Genome match |
| Col-0 Seedling Rep 3 | ATH1046 | 41,513,265 | 39,541,854 | 95.3 |
| *dne1* Seedling Rep 3 | ATH1047 | 44,749,000 | 42,693,119 | 95.4 |
| *xrn4* Seedling Rep 3 | ATH1048 | 39,186,115 | 37,134,824 | 94.8 |
| *dne1 xrn4* Seedling Rep 3 | ATH1049 | 41,876,730 | 39,853,410 | 95.2 |
| Col-0 Seedling Rep 4 | ATH1050 | 46,397,807 | 44,223,338 | 95.3 |
| *dne1* Seedling Rep 4 | ATH1051 | 44,733,523 | 42,498,788 | 95.0 |
| *xrn4* Seedling Rep 4 | ATH1052 | 50,500,043 | 48,042,392 | 95.1 |
| *dne1 xrn4* Seedling Rep 4 | ATH1053 | 45,254,280 | 43,038,091 | 95.1 |
| Col-0 Seedling Rep 5 | ATH1054 | 42,072,897 | 40,121,339 | 95.4 |
| *dne1* Seedling Rep 5 | ATH1055 | 38,719,256 | 36,987,712 | 95.5 |
| *xrn4* Seedling Rep 5 | ATH1056 | 65,419,868 | 62,066,672 | 94.9 |
| *dne1 xrn4* Seedling Rep 5 | ATH1057 | 46,505,424 | 44,348,533 | 95.4 |

**Supplemental Table 2. Primers used in the study**

| **Primer** | **Sequence (5' to 3')** | **Notes** |
| --- | --- | --- |
| RT-PCR Analysis | |  |
| 6958 | GGAGAATTGTCCCGTTCCTAG | DNE1 / AT2G15560 Fwd |
| 6959 | AGACTCCTCCATTGGTAGTTACC | DNE1 / AT2G15560 Rev |
| 5100 | GGAAATGGCTTTATATCTACTGACG | XRN4 / AT1G54490 Fwd |
| 5186 | AGTTGATGACTGATCCCTCATCC | XRN4 / AT1G54490 Rev |
| 7027 | TGCCAATCTACGAGGGTTTC | ACT2 / AT3G18780 Fwd |
| 7028 | GTCAGCGATACCTGAGAACATAG | ACT2 / AT3G18780 Rev |
| qRT-PCR Analysis | |  |
| 7060 | ACCGAACATTATCTCTCCTTCTTC | DNE1 / AT2G15560 Fwd |
| 6959 | AGACTCCTCCATTGGTAGTTACC | DNE1 / AT2G15560 Rev |
| 7368 | ACTTGGACTGCCCTTGTATC | PAO2 / AT2G43020 3' Fwd |
| 7287 | GCTCAAACGTTACGCCAATC | PAO2 / AT2G43020 3' Rev |
| 7356 | TACTAGCATCCCGATTCCATTTC | RCC1-Like / AT5G11580 3' Fwd |
| 7357 | CTCTCTCCATCTCCAAGTCCTA | RCC1-Like / AT5G11580 3' Rev |
| 7422 | TCGGTCAAGGAGGAAGAAGTTTCGTTGC | HB-6 / AT2G22430 3' Fwd |
| 7407 | TGCGGCGGCGAATGAAGAAGCCGCCG | HB-6 / AT2G22430 3' Rev |
| 5438 | TGGCTTGGGACTTTTGACA | RAP2.4 / AT1G78080 3' Fwd |
| 5439 | CTTGTACGCCGCCTTATCAT | RAP2.4 / AT1G78080 3' Rev |
| 5329 | CAGGTATCGCTGACCGTATGAG | ACT2 / AT3G18780 Fwd |
| 5330 | CATCTGCTGGAATGTGCTGAGG | ACT2 / AT3G18780 Rev |
| Probes for RNA blot analysis | |  |
| 7284 | GGAGTGAAGGTAACGACAGAAA | PAO2 / AT2G43020 3' Fwd |
| 7285 | CAGAACTCGCATCCTACAATCT | PAO2 / AT2G43020 3' Rev |
| 7286 | TGGACGAGTTCACACTGATTAC | PAO2 / AT2G43020 5' Fwd |
| 7287 | GCTCAAACGTTACGCCAATC | PAO2 / AT2G43020 5' Rev |
| 7304 | GGATTAGGATTATGCCCCGATGTCAA | RCC1-Like / AT5G11580 3' Fwd |
| 7305 | ATCAGCTGACTCTTATCAGCACTCTCCA | RCC1-Like / AT5G11580 3' Rev |
| 7356 | TACTAGCATCCCGATTCCATTTC | RCC1-Like / AT5G11580 5' Fwd |
| 7371 | TCCTTCCTGTAGGTGAGAAGAG | RCC1-Like / AT5G11580 5' Rev |
| 7304 | GGATTAGGATTATGCCCCGATGTCAA | RCC1-Like / AT5G11580 3' Fwd |
| 7305 | ATCAGCTGACTCTTATCAGCACTCTCCA | RCC1-Like / AT5G11580 3' Rev |
| 7404 | CTGAGTGGAGAAGAAGCTTGTGAA | HB-6 / AT2G22430 3' Fwd |
| 7417 | TCCACAGAGAAGAAAAGAGCATCTAATCCA | HB-6 / AT2G22430 3' Rev |
| 5383 | CGAGATCGATTGGGATTCGATTC | RAP2.4 / AT1G78080 3' Fwd |
| 6442 | ACTGTGGCATGGTCGAGCCTGACA | RAP2.4 / AT1G78080 3' Rev |
| 6958 | GGAGAATTGTCCCGTTCCTAG | DNE1 / AT2G15560 Fwd |
| 6959 | AGACTCCTCCATTGGTAGTTACC | DNE1 / AT2G15560 Rev |
| 7027 | TGCCAATCTACGAGGGTTTC | ACT2 /AT3G18780 3' Fwd |
| 7028 | GTCAGCGATACCTGAGAACATAG | ACT2 /AT3G18780 3' Rev |

| Cloning 35S:CFP:DNE1^WT^ and 35S:CFP:DNE1^D153A^ | |  |
| --- | --- | --- |
| 7033 | GCTGTACAAGgggcccATGATACAAAACGCTATGTC | DNE1 / AT2G15560 Fwd (BsrGI) |
| 7034 | actagtTTAAACCGGGCTGATGAGTTTC | DNE1 / AT2G15560 Rev (SpeI) |
| 7209 | TTGGGGCCCATGATACAAAACGCTATGTC | DNE1 / AT2G15560 Fwd (PspOMI) |
| 7212 | CAATATATGAAGCGCAGGCGCGAAATCAACAgCACCCGATAC | DNE1 / AT2G15560 Rev (D153A) |
| 7213 | CCGCCTGCGACTATCGTTCTCGTATCGGGTGcTGTTGATTTCGC | DNE1 / AT2G15560 Fwd (D153A) |
| 7214 | GGGAGGCCTGGATCGACTAGtTTAAACCGGG | DNE1 / AT2G15560 Rev (SpeI) |
| Genotyping PCR | |  |
| 6960 | GCAAGGAGCTTACATTGCTTG | DNE1 / AT2G15560 Rev |
| 6961 | GCGACTATCGTTCTCGTATCG | DNE1 / AT2G15560 Fwd |
| 5100 | GGAAATGGCTTTATATCTACTGACG | XRN4 / AT1G54490 Fwd |
| 5186 | AGTTGATGACTGATCCCTCATCC | XRN4 / AT1G54490 Rev |
| 1557 | TGGTTCACGTAGTGGGCCATCG | T-DNA LBa1 for *dne1-1* |
| 1521 | TAGCATCTGAATTTCATAACCAATCTCGATACAC | T-DNA LBB3 for *xrn4-5* |
| Modified 5' RLM- RACE | |  |
| 4402 | GTTCAGAGTTCTACAGTCCGAC | 5' Adaptor (1st reaction) |
| 4404 | AATGATACGGCGACCACCGACAGGTTCAGAGTTCTACAGTCCGA | 5' Adaptor (2nd reaction) |
| 7285 | CAGAACTCGCATCCTACAATCT | PAO2 / AT2G43020 3' Rev (1st reaction) |
| 7289 | CCTAGGTCGTTGATTGCTTCT | PAO2 / AT2G43020 3' Rev (2nd reaction) |
| 7305 | ATCAGCTGACTCTTATCAGCACTCTCCA | RCC1-Like / AT5G11580 3' Rev (1st reaction) |
| 7372 | CTAGCCGTTTAATCTCCTCAGTT | RCC1-Like / AT5G11580 3' Rev (2nd reaction) |
| GMUCT v2 | |  |
| 7343 | GUUCAGAGUUCUACAGUCCGACGAU**CAGCAG** | 5' RNA Adapter with EcoP15I site in bold |
| 7344 | CTGGAGTTCAGACGTGTGCTCTTCCGATCTNNNNNN | GMUCT RA3 |

**Supplemental Table 3. Overrepresented GO terms among DNE1 targets.** Significant GO Biological Processes were identified using the Biomaps tools (Virtual Plant), and the Fisher Exact Test (with FDR correction) was used to calculate their P-values (<0.05).

| **Term** | **GO Term ID** | **P-value** |
| --- | --- | --- |
| glucosinolate metabolic process | GO:0019760 | 0.0002 |
| regulation of RNA metabolic process | GO:0051252 | 0.0028 |
| regulation of biological process | GO:0050789 | 0.0031 |
| regulation of RNA biosynthetic process | GO:2001141 | 0.0055 |
| regulation of cellular metabolic process | GO:0031323 | 0.0065 |
| regulation of cellular process | GO:0050794 | 0.0077 |
| regulation of nucleobase-containing compound metabolic process | GO:0019219 | 0.0081 |
| biological regulation | GO:0065007 | 0.0137 |
| regulation of cellular biosynthetic process | GO:0031326 | 0.0171 |
| regulation of macromolecule biosynthetic process | GO:0010556 | 0.0189 |
| RNA metabolic process | GO:0016070 | 0.0212 |
| regulation of biosynthetic process | GO:0009889 | 0.0216 |
| response to abscisic acid | GO:0009737 | 0.0266 |
| DNA-templated transcription | GO:0006351 | 0.0274 |
| RNA biosynthetic process | GO:0032774 | 0.0311 |
| aromatic compound biosynthetic process | GO:0019438 | 0.0315 |
| response to alcohol | GO:0097305 | 0.0316 |
| secondary metabolic process | GO:0019748 | 0.0331 |
| response to lipid | GO:0033993 | 0.0374 |
| organic cyclic compound biosynthetic process | GO:1901362 | 0.0404 |
| response to hormone | GO:0009725 | 0.0475 |

**Supplemental Table 4. G-quadruplex prediction within DNE1 target sequences.** DNE1 target sequences that passed the threshold values for G4 prediction scoring (G4 RNA Screener web interface) are shown. Cleavage site sequences used in the analysis are from Data Set 1. Three prediction scoring systems and their threshold values (in parenthesis) that describe the likelihood of G4 observation used in this analysis are cGcC, consecutive G on consecutive C ratio (>4.5); G4H, G4 Hunter (>0.9); and G4NN, G4 Neural Network (0.5). Values that are higher than threshold score are highlighted.

| **description** | **sequence** | **cGcC** | **G4H** | **G4NN** |
| --- | --- | --- | --- | --- |
| AT5G22830.1 | CAGUAAUAGUAGUGAUGGAGAUAACGGAGGAGGAAGAGAU | 11.5 | 0.5 | 0.5 |
| AT1G24625.1 | UCAGGUGGUUGAGGAAGCUGAGGCUCCGGUGGUUGUGGUG | 4.7 | 0.7 | 0.5 |
| AT4G38470.2 | UACGGGAUUGUGCUAUGGGAGUUGUUGACUGGGAAGCUUC | 7.2 | 0.7 | 0.4 |
| AT3G01690.1 | AGGUAGUGGAUUGUUCUCAUGGGAAACAACUAUGGGAACU | 6.0 | 0.6 | 0.2 |
| AT5G63160.3 | UAGAGGUGAAAAGGACAAGAAAAUGGUUGAGGACACGAAG | 7.3 | 0.5 | 0.2 |
| AT4G37000.1 | GAUUGAUGCGGAGGAAGAGGAUAAGUUGGAGGAGAUAUUG | 27.0 | 0.7 | 0.2 |
| AT5G05250.1 | CGGGGUAAAAUUAAUGGCGUAUGCAAAGAUCGGUUACGGU | 7.0 | 0.7 | 0.2 |
| AT5G24930.1 | GGGAGUGGUGCCAGAUGGUGGUUCGGUGGCUGAUGUAUCG | 5.1 | 0.7 | 0.2 |
| AT5G03380.2 | AUUUGGUUAUAGUUAAGGGGAUCAUUGACGUGAAACAACU | 7.0 | 0.5 | 0.2 |
| AT4G36540.1 | AAAGAGUUGAGAGUUACUUGGGAAACAACAAUGAUGAGCU | 4.5 | 0.3 | 0.2 |
| AT1G78100.1 | UGGAGGUGGAAUUAUACGGUGGUGAUGUCAAGCUUGAGAA | 8.3 | 0.6 | 0.1 |
| AT1G22190.1 | GUCGGAGCUGACGUUUGGUGAUACGGAGGAGGAGAUUCAG | 5.4 | 0.6 | 0.1 |
| AT5G53500.1 | AAGAAGUGGUCAGUGGGAGAGGUUUCAAUCGGUUGAUGUA | 9.3 | 0.6 | 0.1 |
| AT1G53870.4 | UCAAAAGAAACUGGGAUUCUUCAAAUCUUGGGAAGGAUAU | 5.0 | 0.5 | 0.1 |
| AT1G53890.3 | UAUAUAGAAACUGGGAUUCUUCAAAUCUUGGGAAGGAUAU | 6.3 | 0.5 | 0.0 |
| AT4G24900.2 | GCUUGAUGCAAAUGAUGGGGAUUUUUCUAGUGUUCAACUA | 5.2 | 0.4 | 0.0 |
| AT2G42190.3 | AGAAGGAAAAAAAGAAACUUUAUACUGUUUGGUUCUUAGG | 5.0 | 0.3 | 0.0 |
| AT1G60190.1 | UGAAUCUGUGUCAUAACGGCGGAAGUGACGUCGUUGGGUC | 3.6 | 0.4 | 0.7 |
| AT2G13360.2 | GAUGAUACUGAAGGAUGUUGGAUACCCAGUUGUAAUGGGA | 2.2 | 0.3 | 0.7 |
| AT2G47450.1 | CAUGGCGGCUGGUUACGUGAGGCCGGAGGUGGUGGAGGCG | 4.3 | 0.8 | 0.7 |
| AT1G30200.1 | AAUCUGCAGGCAUGGAGGAUGCCGAAGGGAGGUUCUCUGA | 3.3 | 0.5 | 0.6 |
| AT1G58807.2 | UGGCCAUGGCGAAGAAGGGUGACCAGUUGGAAGGUGCAUA | 3.1 | 0.5 | 0.5 |
| AT1G59124.2 | GGCCAUGGCGAAGAAGGGUGACCAGUUGGAAGGUGCAUAC | 2.8 | 0.5 | 0.5 |
